# Herpesviral G Protein-Coupled Receptors Activate NFAT to Induce Tumor Formation via Inhibiting the SERCA Calcium ATPase

**DOI:** 10.1371/journal.ppat.1004768

**Published:** 2015-03-26

**Authors:** Junjie Zhang, Shanping He, Yi Wang, Kevin Brulois, Ke Lan, Jae U. Jung, Pinghui Feng

**Affiliations:** 1 Department of Molecular Microbiology and Immunology, Norris Comprehensive Cancer Center, Keck School of Medicine, University of Southern California, Los Angeles, California, United States of America; 2 Institut Pasteur of Shanghai, Chinese Academy of Sciences, Shanghai, P.R. China; Rosalind Franklin University of Medicine and Science, UNITED STATES

## Abstract

G protein-coupled receptors (GPCRs) constitute the largest family of proteins that transmit signal to regulate an array of fundamental biological processes. Viruses deploy diverse tactics to hijack and harness intracellular signaling events induced by GPCR. Herpesviruses encode multiple GPCR homologues that are implicated in viral pathogenesis. Cellular GPCRs are primarily regulated by their cognate ligands, while herpesviral GPCRs constitutively activate downstream signaling cascades, including the nuclear factor of activated T cells (NFAT) pathway. However, the roles of NFAT activation and mechanism thereof in viral GPCR tumorigenesis remain unknown. Here we report that GPCRs of human Kaposi’s sarcoma-associated herpesvirus (kGPCR) and cytomegalovirus (US28) shortcut NFAT activation by inhibiting the sarcoplasmic reticulum calcium ATPase (SERCA), which is necessary for viral GPCR tumorigenesis. Biochemical approaches, entailing pharmacological inhibitors and protein purification, demonstrate that viral GPCRs target SERCA2 to increase cytosolic calcium concentration. As such, NFAT activation induced by vGPCRs was exceedingly sensitive to cyclosporine A that targets calcineurin, but resistant to inhibition upstream of ER calcium release. Gene expression profiling identified a signature of NFAT activation in endothelial cells expressing viral GPCRs. The expression of NFAT-dependent genes was up-regulated in tumors derived from tva-kGPCR mouse and human KS. Employing recombinant kGPCR-deficient KSHV, we showed that kGPCR was critical for NFAT-dependent gene expression in KSHV lytic replication. Finally, cyclosporine A treatment diminished NFAT-dependent gene expression and tumor formation induced by viral GPCRs. These findings reveal essential roles of NFAT activation in viral GPCR tumorigenesis and a mechanism of “constitutive” NFAT activation by viral GPCRs.

## Author Summary

G protein-coupled receptors (GPCRs) constitute the largest family of proteins that transmit signal across plasma membrane. Herpesviral GPCRs (vGPCRs) activate diverse signaling cascades and are implicated in viral pathogenesis (e.g., tumor development). In contrast to cellular GPCRs that are chiefly regulated via cognate ligand-association, vGPCRs are constitutively active independent of ligand-binding. vGPCRs provide useful tools to dissect signal transduction from plasma membrane receptors to nuclear transcription factors. To probe the activation of nuclear factor of T cells (NFAT), we demonstrate that vGPCRs target the ER calcium ATPase to increase cytosolic calcium concentration and activate NFAT. Inhibition of NFAT activation impairs tumor formation induced by vGPCRs, implying the antitumor therapeutic potential via disabling NFAT activation.

## Introduction

Herpesviruses are ubiquitous pathogens and their infections contribute to a number of malignancies in humans [1]. The lymphotropic gamma herpesviruses, including Kaposi’s sarcoma-associated herpesvirus (KSHV, also known as HHV-8) and Epstein-Barr virus (EBV or HHV-4), are large DNA tumorigenic viruses [2]. Remarkably, these viruses have pirated a number of cellular genes to assist the completion of crucial steps of infection cycle consisting of lytic replication and latent infection. Under immuno-compromised conditions, uncontrolled replication of these viral pathogens results in aberrant cell proliferation that is associated with and underpinned by inflammation [3,4]. Discovered by Yuan Chang, Patrick Moore and their coworkers in 1994, KSHV is the etiological agent of Kaposi’s sarcoma (KS), primary effusion lymphoma (PEL) and multicentric Castleman’s disease (MCD) [5,6]. It is believed that KS is of endothelial origin, whereas PEL and MCD are malignancies of lymphoid cells.

Among genes pirated by human herpesviruses, G protein-coupled receptor (GPCR) is a common target and implicated in viral pathogenesis [7]. All gamma herpesviruses express one GPCR homologue, while genomes of beta-herpesviruses contain up to four copies of GPCR [8,9]. Herpesviral GPCRs activate multiple cellular signaling cascades that collectively contribute to viral infection and pathogenesis[10]. The GPCR homologue of KSHV (designated kGPCR) is capable of activating diverse signaling pathways [11,12]. Prominent examples are the PI3K-AKT axis for cell proliferation [13,14] and pertinent signal pathways leading to the activation of key transcription factors, including NF-κB, NFAT and AP-1 [15,16]. When expressed in transgenic mouse, kGPCR is sufficient to induce KS-like tumors, implying its contribution to the development of human KS [17]. Importantly, kGPCR activates downstream signaling events independent of association with its cognate ligands, which is known as constitutive activity [12]. Previous structural studies pointed to the conformation adopted by the transmembrane helices that enable the constitutive signaling capacity of kGPCR [18]. However, the molecular detail of viral GPCRs in activating specific signaling cascade remains unclear, one of which is the NFAT signaling cascade.

The NFAT family consists of five closely-related members, known as NFAT1-NFAT5. In contrast to NFAT5 that is regulated by osmotic stress [19,20], the other four NFAT proteins are activated by elevated cytosolic calcium concentration [21,22]. Structurally, NFAT proteins contain an amino-terminal transactivation domain, a regulatory domain, a DNA-binding domain and a carboxyl-terminal domain [22]. The DNA-binding domain belongs to the large family of Rel-homology domain (RHD) that was originally characterized in NF-κB members. The regulatory domain consists of multiple serine-rich sequences that are phosphorylated by several kinases, including casein kinase 1 (CK1), glycogen synthase kinase 3 (GSK3) and dual-specificity tyrosine-phosphorylation-regulated kinase (DYRK) in resting cells [23–26]. When cells are activated by surface receptors that are coupled to calcium influxes, cytosolic calcium increase enables the activation of calmodulin and diverse calmodulin-dependent enzymes. Calcineurin, a phosphatase of the calmodulin-dependent enzymes, binds to its docking site within the amino-terminal region of NFAT and dephosphorylates serine/threonine residues of the NFAT regulatory domain, resulting in the nuclear translocation and activation of NFAT [21,27]. Nuclear dephosphorylated NFAT up-regulated the expression of diverse genes, including COX-2, RCAN1, IL-8 and ANGPT2 [28–34]. The sarco/endoplasmic reticulum calcium ATPase (SERCA) pumps calcium back to the SR/ER compartment, thereby restoring calcium gradient and cellular resting state [35]. Although viral GPCRs, e.g., KSHV kGPCR and human cytomegalovirus (HCMV) US28, are known to potently activate NFAT [15,16,36], the mechanism of NFAT activation and the contribution thereof to the tumorigenesis of these viral GPCRs remain unclear. Moreover, it is not well understood how NFAT activation impacts KSHV infection and pathogenesis in particular and herpesvirus in general.

We report here that viral GPCRs target the SERCA ATPase to elevate cytosolic calcium and promote NFAT activation. kGPCR expression in endothelial cells installed a gene expression profile of NFAT signature and NFAT-dependent genes were up-regulated in kGPCR-induced mouse lesions and human KS tumors. Uncoupling NFAT activation from kGPCR diminished tumor formation in a xenograft mouse model, indicating the critical roles of NFAT in kGPCR tumorigenesis. Similarly, HCMV US28 interacted with SERCA2 to activate NFAT and NFAT activation is necessary for US28-induced tumor formation. These results unveil a molecular mechanism by which viral GPCRs activate signaling events independent of ligand binding, underpinning the constitutive activity of viral GPCRs in signaling and tumorigenesis.

## Results

### kGPCR Induces NFAT Activation

**Fig 1:**
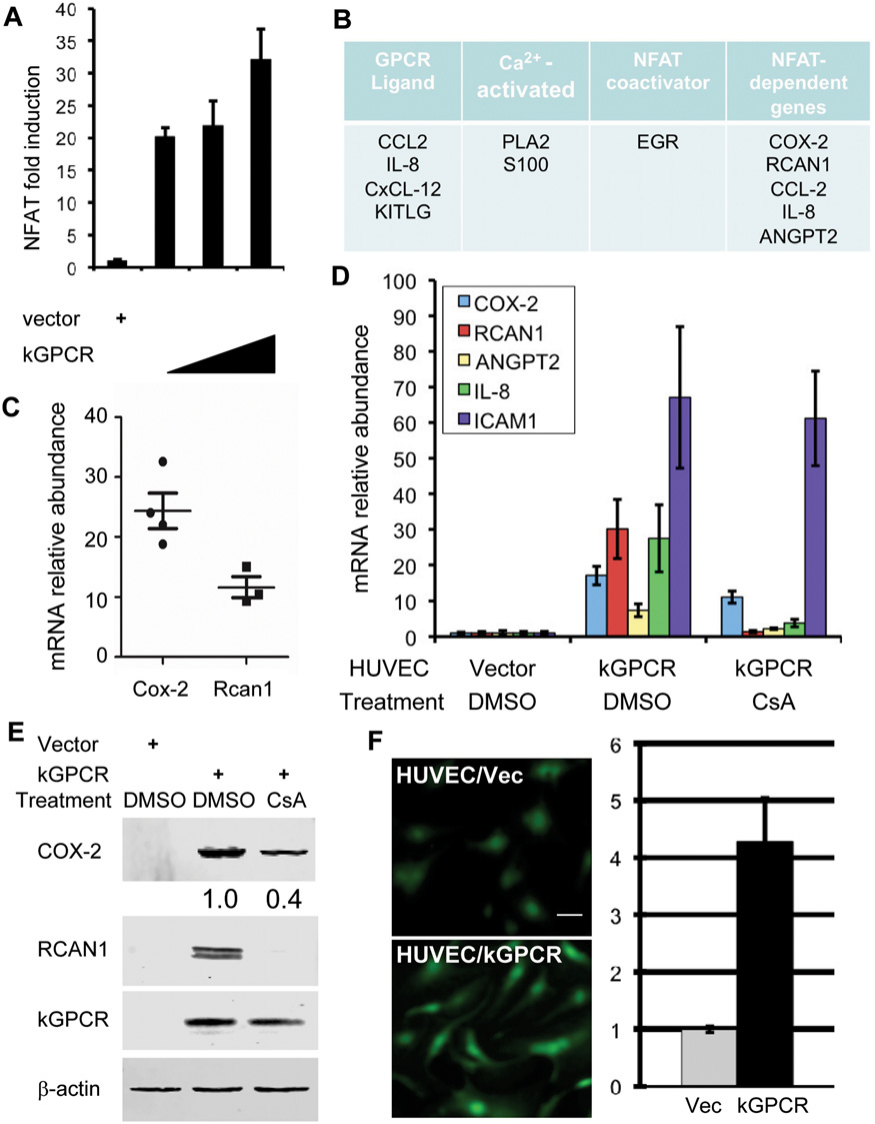
kGPCR activates the NFAT signaling cascade. (A) 293T cells were transfected with the NFAT reporter plasmid cocktail and increasing amount of a plasmid containing kGPCR. NFAT activation was determined by luciferase reporter assays. (B) Top candidates in relation to the NFAT signaling cascades were identified by microarray analysis. (C) Relative mRNA abundance of COX-2 and RCAN1, in relation to β-actin, was calculated from multiple probes of two independent microarray analyses. (D and E) HUVEC cells were mock-treated or treated with cyclosporine A (CsA, 0.5 μM) for 12 h (D). Total RNA was extracted and analyzed by quantitative real-time PCR with primers specific for COX-2, RCAN1, Angiopoietin 2 (ANGPT2), IL-8 and ICAM1. Fold induction of these transcripts in HUVEC/kGPCR cells was calculated in relation to those in control HUVEC cells (D). Whole cell lysates were analyzed by immunoblotting with antibodies against indicated proteins (E) and numbers indicate the relative intensity of COX-2 protein measured in top panel. (F) HUVEC cells were loaded with Fluoro 4-AM and recorded (left panels), and fluorescent intensity was quantified with ImageJ (right panel).

We and others have shown that herpesviral GPCRs induce NFAT activation [16,37,38], despite the roles and mechanism of NFAT activation by these viral GPCRs are not well understood. Using a luciferase reporter, we found that kGPCR expression potently activated NFAT signaling cascades in a dose-dependent manner (Fig. 1A). Moreover, kGPCR expression induced robust nuclear translocation and dephosphorylation of NFAT (S1A–S1C Fig), comparable to ionomycin treatment, while demonstrated no effect on the expression of the catalytic subunit of calcineurin (CnA) (S1D Fig). To examine signaling events downstream of kGPCR in endothelial cell, we performed a genome-wide microarray analysis with human umbilical vein endothelial cells (HUVEC) expressing kGPCR and searched for NFAT-related genes. This analysis uncovered a list of top candidate genes that centered on NFAT signal transduction (Fig. 1B). With a top-down view, we classified these factors according to their link to GPCR, intracellular calcium, NFAT or genes of NFAT-dependent expression (Fig. 1B). Specifically, these included GPCR ligands [CxCL12, CCL2, IL-8 and KIT ligand (KITLG)] [39–42], calcium-dependent effectors [phospholipase A2 (PLA2G4A) and S100 calcium-binding protein] [43,44], NFAT co-activators (EGR) [45], and finally a number of proteins whose expression is up-regulated by NFAT activation [COX-2 (also known as PTGS2), RCAN1, CCL2, IL-8 and angiopoietin 2 (ANGPT2)] [33,46]. Among them, microarray analysis indicated that the expression of COX-2 and RCAN1 were up-regulated by ~24 and 13-fold, respectively (Fig. 1C). These proteins constitute a signaling network that is meshed by key components of the NFAT pathway.

We then selected a few NFAT-dependent genes for quantitative real-time PCR (qRT-PCR) analysis. This analysis showed that, in comparison to control HUVEC, the expression of COX-2 and RCAN1 were up-regulated by ~18 and ~30-fold in kGPCR-expressing HUVEC, respectively (Fig. 1D). The expression of IL-8, ANGPT2 and ICAM1 was also increased by kGPCR (Fig. 1D). Notably, we purposefully selected the NF-κB-dependent ICAM1 transcript for specificity comparison. To further validate the NFAT-dependent expression of these genes, we treated HUVEC/kGPCR cells with cyclosporine A (CsA), a specific pharmacological inhibitor of calcineurin, and determined their expression by qRT-PCR. We found that CsA reduced COX-2 expression by ~50% (Fig. 1D), whereas completely abolished the expression of RCAN1, IL-8 and ANGPT2 (Fig. 1D). The expression of ICAM1 induced by kGPCR was not affected by CsA treatment, consistent with its NF-κB-dependent expression. Immunoblot analysis further confirmed the elevated protein levels of COX-2 and RCAN1 that were reduced or abolished by CsA treatment, respectively (Fig. 1E). One key step of NFAT activation is the elevation of intracellular calcium concentration. Thus, we assessed cytosolic calcium with a fluorescent probe in kGPCR-expressing HUVEC cells. This analysis showed that kGPCR expression increased cytosolic calcium by more than four-fold, compared to control HUVECs (Fig. 1F). Together, these results show that kGPCR activates NFAT to influence host gene expression.

### kGPCR-Induced NFAT Activation Is Resistant to Inhibitors That Targets Steps Upstream of ER Calcium Release

**Fig 2:**
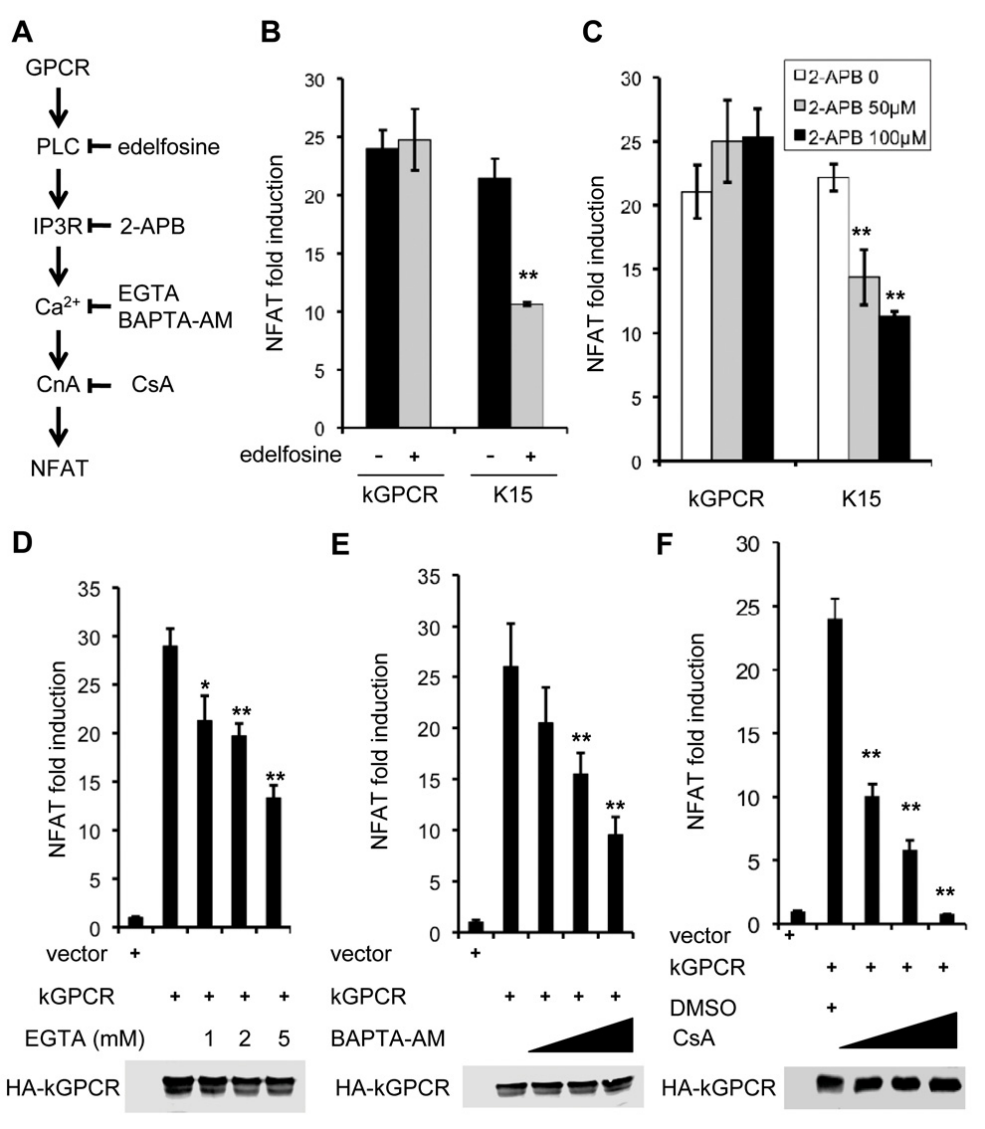
NFAT activation induced by kGPCR is resistant to inhibitors targeting components upstream of ER calcium release. (A) Schematic diagram showing key components and their corresponding inhibitors of the GPCR-NFAT pathway. (B-F) HEK293T cells were transfected with an NFAT reporter cocktail and a plasmid containing KSHV GPCR or K15. At 6 h post-transfection cells were treated with the indicated inhibitors in (B, C and F), or solvent (ethanol for C and DMSO for D and F). For D and E, at 24 h post-transfection, cells were treated with EGTA or BAPTA-AM for 5 h and NFAT activation was determined by luciferase reporter assay. Whole cell lysates were analyzed by immunoblotting with anti-HA (kGPCR) antibody (D, E and F).

To dissect the regulation of NFAT activation by kGPCR, we used pharmacological inhibitors that target key components of the GPCR-NFAT pathway (Fig. 2A). These include inhibitors of phospolipase C [edelfosine] and IP3 receptor [2-aminoethoxydiphenyl borate (2-APB)], chelators to deplete extracellular (EGTA) or intracellular (BAPTA-AM) calcium, and CsA to block calcineurin. Reporter assays indicate that kGPCR-induced NFAT activation was not impacted by edelfosine and 2-APB, inhibitors of PLC and IP3R, respectively (Fig. 2B and C). By contrast, calcium chelators (EGTA and BAPTA-AM) and CsA significantly inhibited NFAT activation induced by kGPCR (Fig. 2D-F). Under the same conditions, edelfosine and 2-APB impaired NFAT activation induced by K15 that did so in a PLC-dependent manner [47](Fig. 2B and C). Importantly, the treatment with these pharmacological inhibitors did not impact cell viability (S2A–S2E Fig). These results, together with the increased cytosolic calcium by kGPCR, suggest that kGPCR increases intracellular calcium independent of PLC, pointing to the step of calcium release.

### kGPCR Interacts with SERCA2 and Inhibits SERCA2 ATPase Activity

**Fig 3:**
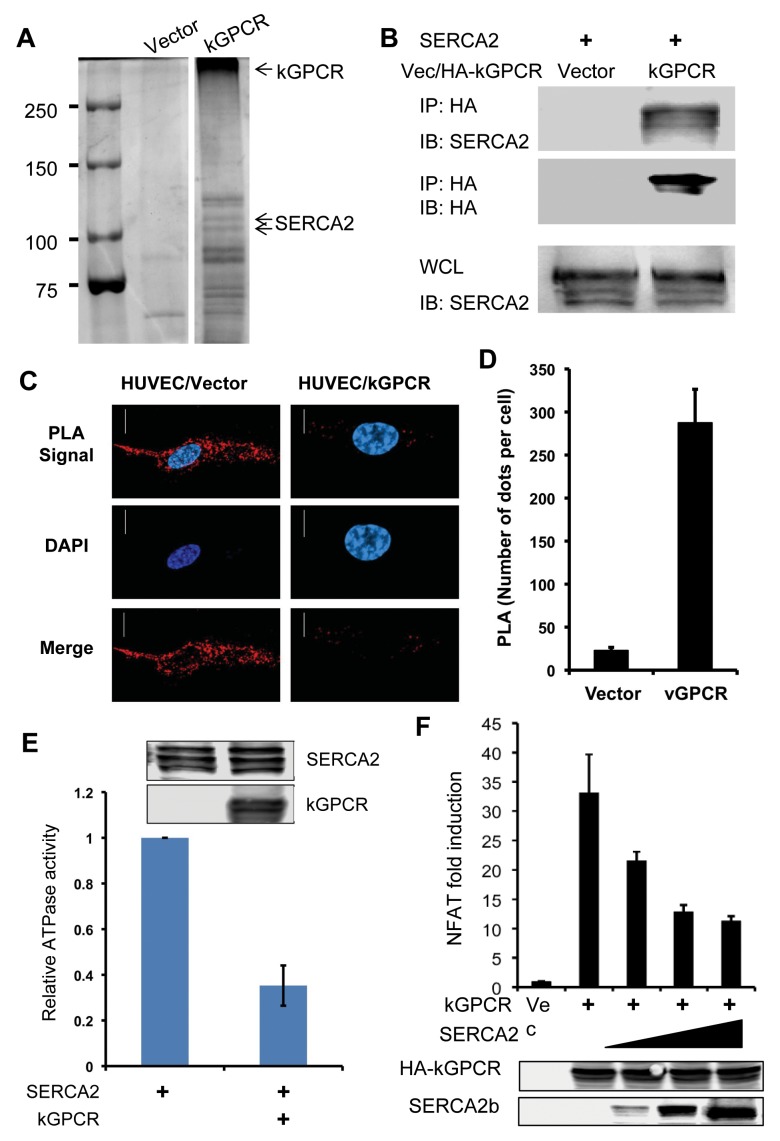
kGPCR interacts with SERCA2 and inhibits its ATPase activity. (A) 293T cells were transfected with a plasmid containing kGPCR. kGPCR was purified with anti-Flag affinity chromatography. kGPCR-interacting proteins were analyzed by commassie staining and photographed, identified by mass spectrometry analysis. A separating line indicates a lane that was removed between lanes of vector and kGPCR. Note, heat-denatured kGPCR oligomerized and migrated slowly. (B) 293T cells were transfected with plasmids containing SERCA2 or kGPCR. Centrifuged cell extract was precipitated with anti-HA (kGPCR). Precipitated proteins and whole cell lysates were analyzed by immunoblot with indicated antibodies. (C and D) HUVEC endothelial stable cells were analyzed by proximity ligation assay (red) and stained with DAPI. Cells were analyzed by confocal microscopy (C). The intensity of proximity ligation was semi-quantitatively determined by counting more than 100 cells (D). (E) 293T cells were transfected with a plasmid containing Flag-tagged SERCA2 without or with a plasmid containing kGPCR. SERCA2 was precipitated with anti-Flag agarose and analyzed by ATPase activity in vitro. Precipitated SERCA2 and whole cell lysates were analyzed by immunoblot (insert). (F) 293T cells were transfected with an NFAT reporter cocktail, a plasmid containing kGPCR and increasing amount of a plasmid containing SERCA2. NFAT activation was determined by luciferase reporter assays.

To identify cellular target(s) that interacts with kGPCR, we performed one-step affinity purification and analyzed kGPCR-binding proteins by mass spectrometry. This approach identified SERCA2 as a major kGPCR-interacting partner (Fig. 3A). Mammalian cells express three isoforms of SERCA, among which SERCA2 is ubiquitously expressed in many tissues. Indeed, SERCA2b and kGPCR were precipitated together from extract of transfected 293T cells (Fig. 3B). Considering that kGPCR and SERCA2 are multi-transmembrane proteins and are not amenable for deletion or truncation analysis, we employed proximity ligation assay (PLA) to assess protein interaction in situ. PLA is devised to detect and localize interacting proteins with single molecule resolution and to be objectively quantified in unmodified cells or tissues [48]. In principle, each single spot of PLA is amplified from one pair of interacting molecules, providing quantitative measurement of physiological protein-protein interactions. As shown in Fig. 3C, fluorescence was barely detected in control HUVEC cells. However, bright fluorescent spots were readily detected in HUVEC cells stably expressing kGPCR. Fluorescent spots were scattered surrounding the nucleus, reminiscent of the intracellular distribution of ER/TGN organelles. Counting more than 100 cells of each group, we found that HUVEC/kGPCR cells yielded >10-fold fluorescent spots than control HUVEC cells (Fig. 3D). These results indicate that kGPCR interacts with SERCA2.

SERCA2 transports calcium from the cytosol into the ER lumen, restoring a calcium gradient between the cytosol and the ER compartment. This active transfer of calcium against gradient by SERCA is powered by and coupled to ATP hydrolysis. To examine the effect of kGPCR on SERCA2, we precipitated SERCA2 from transfected cells and determined the ATPase activity of SERCA2 without or with kGPCR. This assay showed that kGPCR expression reduced the ATP hydrolysis of SERCA2 by more than 60%, indicating that kGPCR inhibits the ATPase activity of SERCA2 (Fig. 3E). Given the opposing activity of kGPCR and SERCA in regulating cytosolic calcium concentration, we then assessed the effect of SERCA2 over-expression on kGPCR-induced NFAT activation. As shown in Fig. 3F, we found that SERCA2 expression reduced kGPCR-induced NFAT activation in a dose-dependent manner, indicating that these two molecules antagonize each other in regulating NFAT activation. Thapsigargin is a pharmacological agent that specifically inhibits SERCA and elevates cytosolic calcium, thereby promoting NFAT activation. We exploited thapsigargin to probe the interaction between kGPCR and SERCA2, given that both kGPCR and thapsigargin inhibit SERCA2 to increase cytosolic calcium. We first examined the effect of thapsigargin on kGPCR-induced NFAT activation. Reporter assays showed that treatment with thapsigargin had no enhancing effect on NFAT activation induced by kGPCR (S3A Fig). Similar result was observed for ionomycin, an ionophore that raises intracellular calcium. These results suggest that kGPCR functions redundantly with thapsigargin and ionomycin in elevating cytosolic calcium and activating NFAT. We next examined kGPCR interaction with SERCA2 with and without thapsigargin. Remarkably, thapsigargin completely abolished kGPCR interaction with SERCA2 (S3B Fig), suggesting that kGPCR and thapsigargin disrupt SERCA2 activity in a similar manner. These findings collectively support the conclusion that kGPCR inhibits SERCA to increase cytosolic calcium and enable NFAT activation.

### HCMV US28, but Not EBV BILF1, Interacts with SERCA2 and Activates NFAT

All gamma herpesviruses encode at least one GPCR, while beta herpesviruses express up to four GPCR homologues. We then examined NFAT activation by GPCR homologues of human EBV (BILF1) and CMV (US28). Reporter assay indicated that, similar to kGPCR, US28 potently activated NFAT in a dose-dependent manner, whereas BILF1 failed to do so (S4A Fig), despite that all three viral GPCRs were expressed at similar levels. To probe the mechanism of US28-mediated NFAT activation, we assessed the interaction between viral GPCRs and SERCA2. When US28 was precipitated from transfected 293T cells, SERCA2 was readily detected, indicating that US28 associates with SERCA2 (S4B Fig). However, BILF1 did not interact with SERCA2 by co-IP assay, agreeing with the observation that EBV BILF1 failed to activate NFAT. These results suggest that the interaction with SERCA2 is crucial for HCMV US28 to activate NFAT.

**Fig 4:**
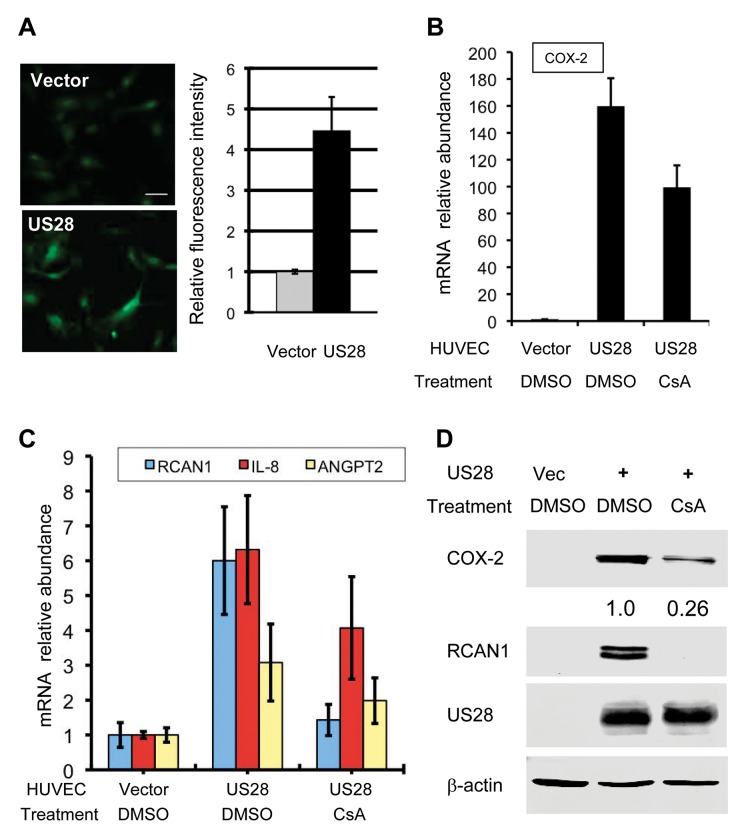
HCMV US28 activates the NFAT signaling cascade. (A) Control HUVEC cells or those stably expressing US28 were loaded with Fluoro 4-AM and recorded (left panels), and fluorescent intensity was quantified with ImageJ (right panel). (B-D) Control HUVEC cells or those stably expressing US28 were treated without or with cyclosporine A (CsA, 0.5 μM) for 12 h and harvested. Total RNA was extracted and cDNA was analyzed by real-time PCR using primers specific for COX-2 (B), RCAN1, IL-8 and angiopoietin 2 (ANGPT2) (C). Whole cell lysates were analyzed by immunoblotting with antibodies against COX-2, RCAN1, US28 and beta-actin (D). Numbers indicate the relative intensity of COX-2 protein measured in top panel.

Next, we measured the relative intracellular calcium concentration in control and US28-expressing HUVEC cells. Fluorescent microscopy and semi-quantitative analysis showed that US28 expression increased intracellular calcium concentration by ~3-fold in HUVEC cells (Fig. 4A). Furthermore, qRT-PCR analysis demonstrated that US28 up-regulated the expression of RCAN1, COX-2, IL-8 and ANGPT2, among which RCAN1 and COX-2 were also confirmed by immunoblotting analysis (Fig. 4B and C). Treatment with CsA reduced the expression of all genes to various extents by qRT-PCR analysis, with completely diminished expression of RCAN1 (Fig. 4B and C). Similar to what was observed for kGPCR, CsA treatment abolished US28-induced RCAN1 protein expression, while partly reduced that of COX-2, as analyzed by immunoblotting (Fig. 4D). Taken together, these findings indicate that HCMV US28, like kGPCR, targets SERCA2 to activate NFAT.

### An EBV BILF1 Chimera Carrying kGPCR Cytoplasmic Loops and Tail Interacts with SERCA2 and Activates NFAT

Despite being a homologue of KSHV kGPCR, EBV BILF1 failed to interact with SERCA2 and activate NFAT. Conventional mutagenesis entailing deletion and truncation is not applicable to the multi-transmembrane GPCR protein. Thus, we explored the chimera strategy to identify sequences that enable vGPCR’s ability to bind SERCA2 and activate NFAT. Previous reports have shown that the cytoplasmic tail is necessary for kGPCR to activate downstream signaling. However, replacing the BILF1 cytoplasmic tail with its counterpart of kGPCR did not confer BILF1 to activate NFAT. Remarkably, when all cytoplasmic loops and tail of BILF1 were replaced with kGPCR equivalents, the BILF1 chimera (designated BILF1c) activated NFAT in a dose-dependent manner (S4C Fig). NFAT activation by BILF1c was not as robust as kGPCR, suggesting that other elements of kGPCR (e.g., transmembrane helices, extracellular N-terminus and loops) also contribute to NFAT activation by kGPCR. In support of the ability of BILF1c to activate NFAT, co-IP assay showed that BILF1c interacted with SERCA2 in transfected 293T cells (S5A Fig). Moreover, NFAT activation by BILF1c was inhibited by calcium chelators (EGTA and BAPTA-AM) and CsA (S5B-D Fig), supporting the conclusion that BILF1c increases cytosolic calcium to activate NFAT. Collectively, these results show that the cytoplasmic loops and tail of kGPCR endow EBV BILF1 to target SERCA2 via physical interaction and that targeting SERCA2 by vGPCRs is sufficient to enable NFAT activation.

### kGPCR Activates NFAT during KSHV Lytic Replication

**Fig 5:**
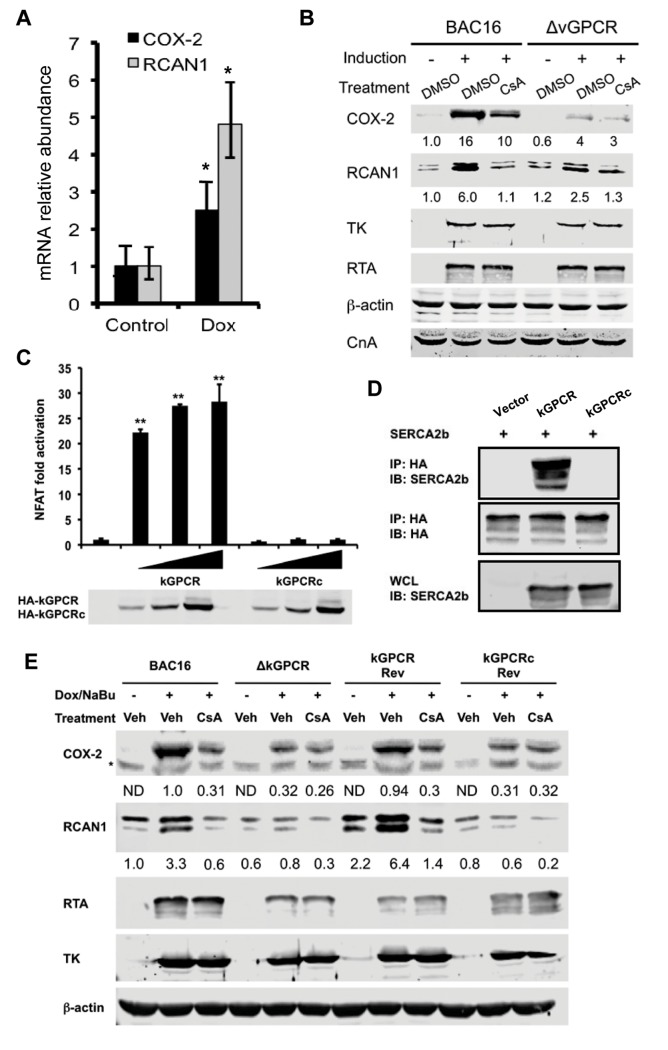
kGPCR activates NFAT during KSHV lytic replication. (A) iSLK.219 cells were induced with doxycycline (0.5 μg/ml) for 48 h and total RNA was extracted, analyzed by qRT-PCR with primers specific for COX-2 and RCAN1. (B) iSLK containing wild-type BAC16 or BAC16 with kGPCR deletion (ΔkGPCR) were induced with doxycycline (0.5 μg/ml) and sodium butyrate (1 mM) for 48 hours, without or with cyclosporine A (CsA, 0.5 μM). Whole cell lysates (WCL) were analyzed by immunoblot with indicated antibodies (B). TK: thymidine kinase (ORF21). (C) 293T cells were transfected with the NFAT luciferase reporter cocktail and increasing amount of a plasmid containing wild-type kGPCR or kGPCR chimera (kGPCRc) in which the cytoplasmidc tail was replaced with the EBV BILF1 counterpart. NFAT activation was determined by luciferase reporter assay at 30 hours post-transfection. (D) 293T cells were transfected with plasmids containing SERCA2b, kGPCR or kGPCR chimera (kGPCRc). WCLs were precipitated with anti-HA (kGPCR or kGPCRc). Precipitated proteins and WCLs were analyzed by immunoblot with indicated antibodies. (E) iSLK cells containing wild-type BAC16 (wild-type), kGPCR-deficient (ΔkGPCR), revertant with wild-type kGPCR (kGPCR Rev) or the kGPCR chimera (kGPCRc Rev) were induced for lytic replication and NFAT activation was inhibited with cyclosporine A as described in (A). WCLs were analyzed by immunoblot with indicated antibodies.

kGPCR is expressed predominantly in the lytic phase. We reasoned that KSHV lytic replication up-regulates NFAT-dependent genes. In iSLK.219 cells that KSHV lytic cycle was induced with doxycycline, mRNAs of COX-2 and RCAN1 were increased to ~3- and 4.5-fold at 48 hours post-induction (Fig. 5A). The recently reported KSHV BAC16 system provided a tool to efficiently induce KSHV lytic replication [49]. To examine the roles of kGPCR in NFAT activation during KSHV lytic replication, we have employed the BAC16 genetic system and engineered a kGPCR-deficient recombinant KSHV (S6A Fig). When iSLK cells harboring wild-type BAC16 were induced by RTA expression and sodium butyrate, a histone deacetylase inhibitor, we found that COX-2 and RCAN1 proteins greatly increased at 48 hours post-induction (Fig. 5B). Importantly, treatment with the NFAT-specific CsA significantly reduced COX-2 induction, while completely abolished RCAN1 protein induction. In iSLK cells harboring kGPCR-deficient BAC16, KSHV lytic replication marginally increased the protein of COX-2 and RCAN1. Moreover, CsA treatment minimally reduced COX-2 and RCAN1 protein, agreeing with the minimal NFAT activation, if any, in the absence of kGPCR. Taken together, kGPCR expression and downstream NFAT activation are responsible for the induced expression of COX-2 and RCAN1 in KSHV lytic replicating cells.

To corroborate the NFAT-dependent gene expression induced by kGPCR in KSHV lytic replication, we constructed recombinant KSHV that kGPCR deletion was restored with wild-type kGPCR or a kGPCR chimera in which the cytoplasmic tail was replaced with the EBV BILF1 counterpart (S6A-C Fig). Consistent with that the cytoplasmic tail of kGPCR is critical for NFAT activation, the kGPCR chimera failed to activate NFAT by reporter assay (Fig. 5C). Moreover, co-immuno-precipitation assay indicated that the kGPCR chimera did not interact with SERCA2, but wild-type kGPCR did (Fig. 5D). Recombinant KSHV BAC16 DNA carrying wild-type, kGPCR revertant and kGPCR chimera were obtained via homologous recombination. Gel electrophoresis confirmed that deletion of kGPCR gene reduced the size of the targeted fragment by ~1 kb analyzed by digestion with both *Kpn*I and *Sbf*I, and the size of the targeted fragment was restored in BAC16 revertant of kGPCR or kGPCRc (S6A-C Fig). The kGPCR loci of BAC16 wild-type, kGPCR deletion, revertant with wild-type kGPCR or kGPCR chimera were further validated by PCR amplification and subsequent sequencing of the PCR products (S6C Fig).

We then transfected KSHV BAC16 DNA and its derivatives into iSLK cells and cells stably carrying KSHV were selected with hygromycin. KSHV lytic replication was reactivated with sodium butyrate and doxycycline to induce RTA expression. In cells that KSHV lytic replication was induced, we also inhibited NFAT activation with cyclosporine A. Using BAC16 wild-type as the positive reference and BAC16ΔkGPCR as the negative reference, we found that the kGPCR revertant demonstrated nearly identical expression of COX-2 and RCAN1 of wild-type BAC16 that were induced by KSHV lytic replication and inhibited by cyclosporine A. By contrast, BAC16 revertant with kGPCR chimera essentially replicated the phenotype in COX-2 and RCAN1 expression of BAC16ΔkGPCR (Fig. 5E). When KSHV early lytic gene products, including RTA and thymidine kinase (TK or ORF21), were examined, no significant difference of these viral proteins were detected among these recombinant KSHV carrying various kGPCR mutants. Consistent with kGPCR-dependent NFAT activation, we also observed NFAT1 dephosphorylation in KSHV replicating iSLK cells in a kGPCR-dependent manner (S6D Fig). Thus, kGPCR expression and consequent NFAT activation during KSHV lytic replication are important for the expression of COX-2 and RCAN1, two NFAT-dependent cellular genes.

### kGPCR Also Activates NFAT via a Paracrine Mechanism

**Fig 6:**
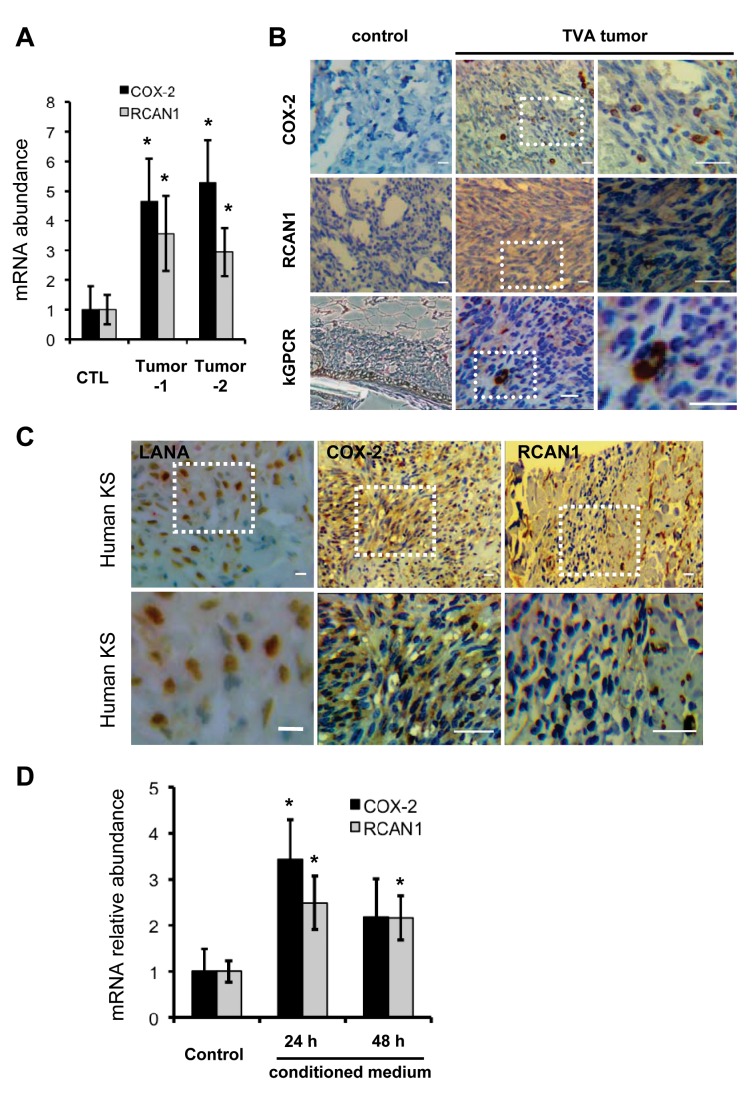
NFAT activation in mouse KS-like lesions and human KS tumors. (A) Total RNA was extracted from normal skin or tumors of tva-kGPCR mice. cDNA was prepared and analyzed by qRT-PCR with primers for COX-2 and RCAN1. (B) Normal skin and tva-kGPCR mouse tumors were analyzed by immunohistochemistry (IHC) staining for COX-2, RCAN1 and kGPCR. Boxed region was amplified and shown on the right. (C) Human KS tumors were analyzed by IHC with antibodies against LANA (left panels), COX-2 (middle panels) and RCAN1 (right panels). Boxed region was amplified and shown at the bottom of each panel. (D) HUVEC cells were incubated with conditioned medium from HUVEC/Vec (control) or HUVEC/kGPCR cells for indicated time. Total RNA was extracted and analyzed by qRT-PCR with primers for COX-2 and RCAN1. Data are presented as mean±SEM. *p<0.05; scale bars: 10 μm.

Although the major constituent of KS tumors is the KSHV latently-infected spindle cell, lytic replicating cells were invariably observed in KS tumors. The *TIE2*-tva mouse model that kGPCR expression in endothelial cells is sufficient to induce KS-like tumors provides a useful tool to recapitulate the genesis of Kaposi’s sarcoma [17]. We examined NFAT-dependent gene expression in tumor tissues derived from the *TIE2*-tva mice infected with retrovirus carrying kGPCR. qRT-PCR analysis revealed that mRNAs of COX-2 and RCAN1 were up-regulated by ~4- and 3-fold in kGPCR-tumors compared to control tissues, respectively (Fig. 6A). Immunohistochemistry staining further showed apparent induction of COX-2 and RCAN1 in kGPCR-tumor (Fig. 6B). Based on the expression level of COX-2, two types of cells were identified in kGPCR-tumors. A small subset of cells of high COX-2 expression had round shape and small cytoplasm, which likely represented infiltrated immune cells. The majority of tumor tissue consists of spindle-shaped or other endothelial cells that had lower COX-2 expression, displaying light brown color in the cytoplasm. By contrast, a uniformed RCAN1 staining pattern was observed in most tumor cells, specifically the spindle-shaped endothelial cells. This result indicates the differential expression of NFAT-dependent genes in cell type-specific manner, which signifies distinct roles of NFAT in corresponding tumor constituents. We further examined the expression of these NFAT-dependent genes in human KS tissue. The expression of COX-2 and RCAN1 was apparent in regions that were positive for LANA, the nuclear antigen and marker for KSHV infected cells (Fig. 6C and S7A-C). Specifically, COX-2 was high in cells with relatively small cytoplasm, implying their immune cell identity. In the spindle-shaped cells, heterogeneous COX-2 expression was observed, with majority of these tumor cells demonstrating cytoplasm staining of COX-2. Interestingly, cells with high RCAN1 protein, residing in region proximal to the slit-like structure, were reminiscent of immune cells, while spindle-shaped tumor cells were stained relatively low for RCAN1. These results show that COX-2 and RCAN1 proteins are highly expressed in KS-like mouse lesion and human KS tumors.

Given that kGPCR is expressed within approximately 5% of cells of tumors derived from the *TIE2*-tva mouse, we reasoned that kGPCR can induce NFAT via paracrine stimulation. In fact, a number of factors that were induced by kGPCR, including IL-8, CCL-2 and KITLG, instigate NFAT activation when bound to their cognate receptors on the cell surface, constituting a feed-forward loop that fuels signaling amplification. To test this hypothesis, we collected conditioned medium from control or kGPCR-expressing HUVECs to stimulate fresh HUVECs. We found that conditioned medium from kGPCR-expressing HUVEC modestly up-regulated the expression of COX-2 and RCAN1 to ~3-fold, compared to conditioned medium from control HUVECs (Fig. 6D). However, when anti-IL-8 antibody was added into medium to neutralize IL-8, we observed minimal impact on the mRNA levels of COX-2 and RCAN1 (S7D Fig). This is likely due to other secreted factors that activate NFAT, such as CCL2 and KITLG. Alternatively, exosome-mediated delivery of these factors or kGPCR may be resistant to neutralizing antibodies. Nevertheless, these results support the conclusion that kGPCR also activates NFAT via a paracrine mechanism, in addition to an autocrine mechanism.

### NFAT Activation Is Crucial for Tumor Formation Induced by Viral GPCRs

**Fig 7:**
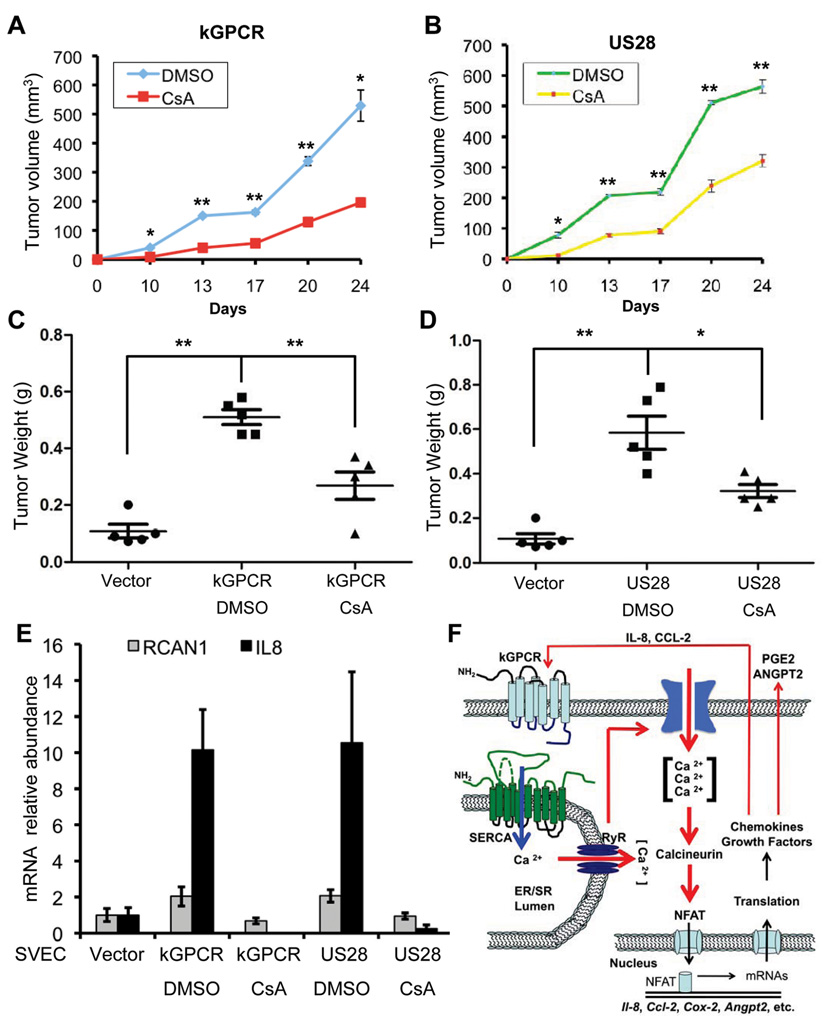
NFAT activation is critical for tumor formation induced by viral GPCRs. (A and B) Age- (6–8 week-old) and sex-matched nude mice (n = 5) were injected subcutaneously with SVEC/kGPCR or SVEC/US28 cells (0.5×10^6^), along with regular SVEC cells (1×10^6^). Tumor volumes were measured twice every week. No tumor was detected for nude mice injected with control SVEC/Vec cells. (C and D) Tumor weight from nude mice (n = 5) was determined when mice were euthanized. Data was presented as Mean ± SED (standard deviation). *p<0.05 and **p<0.01. (E) Total RNA was extracted from tumor tissue and analyzed by real-time PCR with primers specific for RCAN1 and IL-8. (F) A working model concerning players of the NFAT signaling cascades in relaying a feed-forward effect underpinning the tumorigenesis of herpesviral GPCRs. KSHV GPCR (kGPCR) interacts with and inhibits SERCA calcium ATPase, thereby increasing cytosolic calcium concentration and activating calcineurin. Dephosphorylation of NFAT by calcineurin enables NFAT nuclear translocation and up-regulates expression of NFAT-dependent genes (e.g., COX-2, IL-8 and RCAN1). Some of which, such as secreted IL-8 and CCL-2, further promote NFAT activation via binding to their cognate receptor (including kGPCR on the cell surface). Data are represented as mean±SEM.

NFAT is crucial for GPCR-mediated gene expression and many of gene products downstream of NFAT are key players in tumorigenesis. Thus, we examined tumor formation in the xenograft mouse model using murine SVEC endothelial cells expressing kGPCR and US28, under the condition that NFAT activation was inhibited with CsA. As expected, SVEC expressing kGPCR and US28 were sufficient to induce tumor formation in nude mice. Tumor volumes were detectable at two weeks post-inoculation and reached ~500–600 mm^3^ at 24 days post-inoculation (Fig. 7A and B). Under the conditions that nude mice were treated with CsA, tumor volume was reduced by ~60 and ~50% for kGPCR and US28 groups, respectively (Fig. 7A and B). When mice were euthanized and tumor weight was determined, we found that CsA treatment reduced tumor weight by ~50% for both groups derived from kGPCR- and US28-expressing cells (Fig. 7C and D). We then quantified the expression of IL-8 and RCAN1, two NFAT-dependent genes, by qRT-PCR using tumor tissues. We found that kGPCR and US28 had similar levels of induction on IL-8 and RCAN1, with IL-8 being more robustly induced than RCAN1 (Fig. 7E). Strikingly, CsA treatment abolished the induction of IL-8 and RCAN1 gene expression by both viral GPCRs. These results support the conclusion that NFAT activation is critical for tumor formation induced by KSHV kGPCR and HCMV US28.

## Discussion

GPCRs constitute the largest family of signaling molecules that regulates nearly every fundamental biological process. Upon ligand binding, cellular GPCRs are coupled to a panel of heterotrimeric G proteins that are composed of a α subunit and a βγ dimer. These small G proteins are activated via guanidine nucleotide exchanging catalyzed by agonist-stimulated GPCRs and relayed to diverse effectors that influence cellular metabolism and proliferation chiefly through regulated gene expression [50]. Some herpesviral GPCRs, e.g., KSHV kGPCR and HCMV US28, demonstrate ligand-independent constitutive activity to instigate signaling cascades that culminate in regulated gene expression [12,51]. We report here that KSHV kGPCR and HCMV US28 bypass the upstream components of NFAT pathway by interacting with and inhibiting the SERCA calcium ATPase, a key negative regulator of NFAT activation. As such, kGPCR and US28 elevated cytosolic calcium and activated signaling events downstream of SERCA. Furthermore, kGPCR expression installed a gene expression profile signature of NFAT activation. Key effectors downstream of NFAT transcription factors were confirmed in human KS tumors and KS-like lesions derived from the tva mouse model. Importantly, pharmacological inhibitors targeting components upstream of ER calcium release had no detectable effect on NFAT activation induced by kGPCR and US28, whereas calcium chelators and the calcineurin inhibitor CsA effectively diminished kGPCR- and US28-induced NFAT activation. Notably, these results do not exclude the possibility that viral GPCRs activate small G proteins and downstream signaling thereof, either with or without cognate agonists. Previous studies have identified structural elements that enable these viral GPCRs to efficiently couple with small G proteins, contributing to the constitutive signaling capacity [18,52]. Although EBV BILF1 is closely-related to kGPCR, BILF1 failed to interact with SERCA2 and activate NFAT. Replacing the cytoplasmic loops and tail of BILF1 with counterparts of kGPCR enabled BILF1 to interact with SERCA2 and activate NFAT. Conversely, replacing the cytoplasmic tail of kGPCR with that of EBV BILF1 resulted in a kGPCR chimera that failed to interact with SERCA2 and activate NFAT. These studies, entailing gain- and loss-of-function experiments, highlight the pivotal role of interaction with and likely inhibition of SERCA2 in NFAT activation and identifies additional structural elements underpinning the constitutive activation of viral GPCRs. Taken together, our study unravels a distinct action of viral GPCRs in activating NFAT independent of agonist association.

NFAT activation is central for the gene expression of a large spectrum of effectors participating in key physiological events such as immune response, development and homeostasis. Dys-regulation of GPCR-NFAT signaling circuitry underpins diverse human diseased conditions ranging from mild inflammatory responses to life-threatening malignancies including cancer [22,53]. Despite that the roles of NFAT signaling cascades in cell differentiation and development are well established, the contribution of NFAT activation in the development and metastasis of various types of cancers is gradually emerging. Initial studies using clinical samples demonstrated that elevated NFAT activation was detected in tumor biopsies from patients with invasive breast carcinoma. Moreover, expression of constitutively active NFAT1 in breast cancer cells promoted migration and invasion [54,55]. Paradoxically, NFAT proteins were shown to serve as tumor suppressors under certain physiological conditions. For example, NFAT4-deficient mice were reported to be more susceptible to develop T cell lymphomas induced by murine leukemia virus SL3-3 than wild-type mice [56]. This is supported by the observation that NFAT inhibits the expression of cyclin-dependent kinase 4 and cyclin A2 [57,58].

NFAT activation was shown to up-regulate the expression of many effectors that have proliferative and transforming activity. Among NFAT downstream effectors that are involved in the development and maintenance of tumor microenvironment, ANGPT2 has been shown to promote tumor growth and angiogenesis [28,29,59]. Disrupting the interaction between ANGPT2 and its TIE2 receptor suppressed tumor growth and angiogenesis [60]. In support of the findings that intimately link IL-8 and COX-2 to tumor angiogenesis [61–63], KSHV infection induces COX-2 expression that enables viral latent infection and angiogenesis thereof [64]. KSHV K15 and vFLIP, indeed, have been implicated in promoting COX-2 expression [65–67]. vFLIP has also been shown to induce cytokine production including IL-8 [68]. Furthermore, COX-2 is crucial for inflammatory cytokine production, angiogenesis and invasion of KSHV-infected cells [67]. Inhibition of COX-2 blocks HCMV replication [69] and leads to a significant reduction of tumor formation induce by HCMV US28 [70]. Similarly, inhibiting COX-2 also impaired cell survival of the KSHV latently-infected PEL cells [71]. Combining inhibitors of COX-2 and NFAT may synergistically ameliorate malignant conditions associated with HCMV and KSHV infection, given that NFAT inhibition can only partially reduce COX-2 expression. RCAN1, a negative feedback regulator of NFAT activation, was implicated in rendering resistance to the development of various cancers in Down’s syndrome patients. Transgenic mice mimicking RCAN1 trisomy showed significant suppression of tumour growth [72]. Surprisingly, knockout of RCAN1 in mouse also inhibited tumor growth due to hyperactivated calcineurin and apoptosis of endothelial cells [73]. These findings suggest that, perhaps, a balanced NFAT activation is critical for tumorigenesis. We demonstrate here that viral GPCRs activate NFAT to install a unique gene expression profile consisting of effectors (e.g., COX-2, RCAN1, IL-8 and ANGPT2) that constitute the autocrine and paracrine circuitries in amplifying the igniting stimulation (Fig. 7F). We noted that the induction of NFAT-dependent transcripts (e.g., COX-2 and RCAN1) varies in HUVEC cells expressing kGPCR and SLK cells infected with replicating KSHV. This likely reflects different viral factors and cellular proteins that impinge on NFAT activation, in addition to kGPCR. Nevertheless, the autocrine and paracrine mechanism of NFAT activation may be applicable to the tumor microenvironment wherein diverse inflammatory effectors and growth-promoting factors signal through GPCRs and NFAT activation. The central role of NFAT activation in these signaling cascades implies that inhibiting NFAT activation will thwart tumor formation induced by viral GPCRs and other oncogenic proteins. The findings that CsA inhibited kGPCR- and US28-mediated tumorigenesis provide a proof-of-principle to target NFAT for antitumor therapy. In an immune competent host, however, approaches that selectively attack tumor tissues while sparing functional immune system are prerequisite to enable the application of an anti-tumor strategy targeting NFAT activation.

## Materials and Methods

### Constructs, Cell Lines and Compounds

If not specified, pcDNA5/FRT/TO (Invitrogen) and pCDH-CMV-EF-Puro (System Bioscience) were used for transient and stable expression of corresponding genes. For protein expression, the HA epitope was inserted upstream or downstream of protein coding sequence. pSF91-K15-IRESGFP was a gift from Dr. Thomas Schulz (Medizinische Hochschule Hannover). pcDEF-HA-US28 was kindly provide by Dr. Liliana Soroceanu (California Pacific Medical Center Research Institute). MSCV-BILF1 was purchased from addgene. pcDNA3.1-SERCA2b and pNFAT1(1–460)-EGFP were kindly provided by Drs. Jonathan Lytton (University of Calgary) and Yousang Gwack (UCLA). pcDNA5/FRT/TO-HA-BILF1c was constructed by replacing the three intracellular loops and C-terminal tail with the counterpart of KSHV GPCR. pcDNA/FRT/TO-HA-kGPCRc was constructed by replacing the C-terminal tail of kGPCR with the counterpart of EBV BILF1.

HEK293T and immortalized murine endothelial cells (SVECs) were maintaind in Dulbecco's modified Eagle's medium (DMEM) containing 10% fetal bovine serum supplemented with 100 U penicillin/streptomycin. iSLK.219 cells were maintained with G418 (250 μg/ml), hygromycin (400 μg/ml) and puromycin (10 μg/ml). iSLK cells were maintained with G418 (250 μg/ml) and puromycin (1 μg/ml). BAC16 and all the mutants were introduced into iSLK cells by using Fugene HD (Promega) transfection and the stable cell lines were maintained with puromycin (1 μg/ml), G418 (250 μg/ml) and hygromycin B (1,200 μg/ml). Human umbilical vein endothelial cells (HUVEC) were purchase from Lifeline Cell Technology and maintained in human endothelial culture medium according to the instructions. To establish stable cell lines, SVECs or HUVECs were infected with lentivirus containing indicated genes and selected with puromycin (1 μg/ml) as described previously [38,74].

Chemicals used in the study include ionomycin (Sigma), cyclosporin A (Cell Signaling), edelfosine (Sigma), 2-Aminoethyl diphenylborinate (Sigma), BAPTA-AM (Abcam), thapsigargin (Sigma)

### Immunoprecipitation and Immunoblotting

Commercial antibodies used in this study include mouse anti-HA monoclonal antibody and agarose (Sigma), mouse anti- β-Actin monoclonal antibody (Abcam), rabbit anti-COX-2 polyclonal antibody (Abcam), rabbit anti-RCAN1 polyclonal antibody (Sigma), mouse anti-SERCA2 (IID8) monoclonal antibody (Santa cruz), mouse anti-calcineurin Aα (Santa cruz). Thymidine kinase (TK) anti-serum was generated by immunizing rabbit with GST fusion protein containing N-terminal (aa1-330) of TK. RTA antibody were kindly provided by Dr. Yoshihiro Izumiya (UC-Davis). Immunoprecipitation and immunoblotting were carried out as described previously [37]. Briefly, cells were harvested and lysed with NP40 buffer (50 mM Tris-HCl [pH 7.4], 150 mM NaCl, 1% NP-40, 5 mM EDTA) supplemented with a protease inhibitor cocktail (Roche). Centrifuged cell lysates were pre-cleared with Sepharose 4B beads and incubated with HA-agarose at 4°C for 4 h. The agarose beads were washed three times with lysis buffer and precipitated proteins were released by boiling with 1×SDS sample buffer at 95°C for 5 min. Immunoblotting analysis was performed with the indicated primary antibodies and proteins were visualized with IRDye800 conjugated secondary antibodies (Licor) using an Odyssey infrared imaging system (Licor).

### Luciferase Reporter Assay

HEK293T cells in 24-well plates were transiently transfected with a reporter cocktail as previously described [74,75]. The reporter cocktail contained 50 ng of the plasmid expressing firefly luciferase under the control of response elements of NFAT and 100 ng of the plasmid expressing β-galactosidase. The reporter cocktail contained 100 ng, 200 ng and 500 ng of plasmid when increasing dose was indicated and all transfections were balanced with empty vector. Cells were harvested at 24 h post-transfection, lysed and centrifuged supernatant was used to measure luciferase and β-galactosidase activity according to the manufacturer’s instruction (Promega). For inhibitors treatment, cells were treated with the inhibitors and cell toxicity of the inhibitors was evaluated by trypan blue staining (Amresco).

### Immunofluorescence and Immunohistochemistry

HEK293T cells were transfected with plasmids containing EGFP-NFAT1(N) and kGPCR. At 24 h post-transfection, cells were treated with 1 μM of ionomycin for 1 h, fixed with 4% paraformaldehyde and permeabilized with 1% Triton X-100. After staining with primary antibody (anti-HA antibody) and secondary antibody (Alexa 568-conjugated goat anti-mouse antibody), cells were analyzed with a Nikon E800M microscope.

For immunohistochemistry staining [76], mouse or human tissue samples were fixed with 10% (vol/vol) formalin solution (Sigma) overnight. Tissue specimens were dehydrated, embedded in paraffin, and cut into 3-μm sections. Tissue sections were analyzed by immunohistochemistry staining with antibodies against COX-2, RCAN1, HA or LANA and DAB substrate kit (Vector Laboratories). Images were visualized with a Nikon E800M microscope equipped with a Nikon DXM1200 digital camera and the Nikon ACT-1 imaging software system.

### Proximity Ligation Assay

Proximity ligation assay (PLA) was performed by using the Duolink *in situ* starter kit (Sigma-Aldrich) according to previous reports [77,78]. Briefly, HUVEC-Vector or kGPCR stable cells were fixed with 4% PFA for 10 min at room temperature, and incubated with DuoLink blocking buffer for 30 min at 37°C. Cells were then reacted with primary antibodies diluted in Duolink antibody diluents for 1 h and then incubated for another 1 h at 37°C with species-specific PLA probes under hybridization conditions. The PLA probes can be hybridized only when they were in close proximity (<40 nm). Ligation was then performed for 30 min at 37°C. After which, a detection solution containing fluorescently labeled oligonucleotides was used to amply the signal for 100 min at +37°C. The signal was detected as a distinct fluorescent dot under fluorescence microscope.

### Antibody Neutralization of IL-8

Conditioned medium from vector or kGPCR-expressing HUVECs were used to stimulate primary HUVECs. Control IgG or IL-8 neutralization antibody (R&D systems) was included in the conditioned medium (0.5 μg/ml) for 24 h. Then, cells were collected for RNA extraction, reverse transcription-PCR and quantitative real-time PCR analysis.

### SERCA ATPase Activity Assay

HEK293T cells were transfected with a plasmid containing Flag-SERCA2b together with a vector or a plasmid containing kGPCR. SERCA2b was precipitated with anti-Flag antibody-conjugated agarose and used for in vitro ATPase assay. The ATPase activity of SERCA2b was determined by using ATPase assay kit according to the manufacturer's instructions (Innova Biosciences). Briefly, the reaction was carried out in a mixture containing 0.5 M of assay buffer, 0.1 M of MgCl_2_, 2 μM of CaCl_2_ and 10 mM of ATP for 30 min at 37°C. Then 50μl of Gold mix was added to stop reactions. After 2 min, 20 μl of stabilizer solution was added and the absorbance was read at 620 nm at 30 min later.

### Measurement of Intracellular Calcium

HUVEC stable cells were loaded with Fluoro-4-AM (Molecular Probes) for 45 min at 37°C. Cells were then washed and further incubated with fresh medium for 20 min. Live cells were analyzed with a Nikon E800M microscope and the fluorescence signal was quantified by ImageJ (NIH).

### Reverse Transcription-PCR and Quantitative Real-Time PCR

To determine the relative levels of the NFAT downstream genes, reverse- transcription PCR and quantitative real-time PCR were performed as previously reported. Briefly, total RNA was extracted from cells using RNAeasy kit (Qiagen). The RNA was digested with DNase I (New England Biolabs) to remove genomic DNA. One microgram of total RNA was used for reverse transcription with Superscript II reverse transcriptase (Invitrogen) according to the manufacturer’s instruction. The abundance of mRNAs was assessed by qRT-PCR using StepONEPlus Real-Time PCR system (Applied Biosystems). Mouse or human β-actin was used as internal controls. The primers were listed in Table S2.

### Generation of kGPCR and kGPCRc Revertant KSHV (BAC16-kGPCR.Rev and BAC16-kGPCRc.Rev

The kGPCR-deficient KSHV was generated by deleting kGPCR coding sequence in bacterial artificial chromosome 16 (BAC16) as previously described [49]. To generate the revertant mutants, we performed the first around of PCR by amplifying a Kan^R^/I-SceI cassette from the pEP-Kan-S plasmid with the following primers: forward primer GTAGATATCTTCAGTGTTGTGTGCGTCAGTCTAGTGAGGTACCTCCAGGATGACGACGATAAGTAGGG and reverse primer TGCGATATCAACCAATTA ACCAATTCTGATTAG. The PCR products were digested with EcoRV and inserted into pcDNA5/FRT/TO-kGPCR or kGPCRc. Then the second around of PCR was performed with the forward primers AAAGGCGTGGCTAAACAACACCTATACTACTTGTTATTG TAGGCCATGTATCCGTATGATGTTCCTGA or AGGCTAGATTAAATTAAGGGGGAAG GGCACGTAGACATCCGCGGGTCAGGTGGACTGGCTAGGCACCCT and reverse primer AGGCTAGATTAAATTAAGGGGGAAGGGCACGTAGACATCCGCGGGCTACG TGGTGGCGCCGGACATGA to get the revertant PCR segments.

The recombination was performed in the GS1783 Escherichia coli strain as previously described [49]. KpnI and SbfI digestion of the BAC16 DNA followed by either conventional agarose gel electrophoresis or pulsed-field gel electrophoresis were used to verify the constructs. In addition, colony PCR and direct sequencing were performed to verify the correct insertion of the revertant mutants.

### Ethics Statement

All animal experiments were carried out according to the National Institutes of Health principles of laboratory animal care and approved by the University of Southern California Institutional Animal Care and Use Committee (IACUC) with permit number A0372.

### Mouse Xenograft Tumor Formation and Treatment

Six to eight-week old athymic (nu/nu) nude mice (Jackson Laboratory) were used for xenograft experiment. KSHV GPCR or HCMV US28 stable SVEC cells (0.5x10^6^) were harvested, washed, resuspended in PBS, mixed with 10^6^ SVEC cells and injected subcutaneously into the flank of nude mice. Mice were treated with CsA (20 μg/g body weight) every the other day via intrapretoneal injection, and monitored for tumor development twice every week. At 4–6 wk after inoculation, mice were euthanized and tumor weight was determined.

## Supporting Information

S1 Fig Expression of kGPCR induces NFAT activation. (A) HEK 293T cells were transfected with plasmids containing EGFP-NFAT1(N) and kGPCR. At 24 h post-transfection, cells were treated with ionomycin (Iono) (1 μM) and then fixed, stained and analyzed by immunofluorescence microscopy. Representative images were shown. Scale bar, 20 μm. (B) 293T cells were transfected with a plasmid containing EGFP-N1(N) without or with a plasmid containing kGPCR. For 293T cells transfected with the plasmid containing EGFP-NFAT1(N), cells were treated with vehicle (DMSO) or ionomycin (1 μM) for 6 hours. Nuclear NFAT1 was counted with fluorescence microscope. (C and D) Transfection and NFAT activation by ionomycin (C) or cyclosporine A (CsA, D) were carried out in (B). Whole cell lysates were analyzed by immunoblotting with indicated antibodies.
(TIF)

S2 Fig Effect of drug treatment on 293T cells. 293T cells were transfected with plasmids of NFAT reporter and that containing kGPCR. At 6 h post-transfection cells were treated with edelfosine (10 μM) (A), 2-APB (50 and 100 μM) (B) or cyclosporine A (CsA, 2, 20 and 200 nM) (C). At 24 hours, cells were treated with EGTA (1–5 mM) (D) or BAPTA-AM (10, 20 and 50 μM) (E) for 5 hours. Cell viability was determined by trypan blue staining.
(TIF)

S3 Fig Thapsigargin (TG) treatment disrupts the interaction between kGPCR and SERCA2. (A) HEK293T cells were transfected with the NFAT reporter cocktail and a plasmid containing KSHV GPCR. At 20 h post-transfection cells were treated with 1μM of TG or ionomycin for 6 h and NFAT activation was determined by luciferase reporter assay. (B) 293T cells were transfected with plasmids containing indicated genes and, 24 h post-transfection, cells were treated with vehicle (DMSO) or TG (1 μM) for 6 h. Co-immunoprecipitation and immunoblot were carried out as in (B). HC, IgG heavy chain.
(TIF)

S4 Fig HCMV US28, but not EBV BILF1, interacts with SERCA2 and activates NFAT. (A) 293T cells were transfected with the NFAT reporter cocktail and increasing amount of plasmids containing KSHV kGPCR, HCMV US28 and EBV BILF1. NFAT activation was determined by luciferase reporter assays. (B) 293T cells were transfected with plasmids containing SERCA2 and US28 or BILF1. Centrifuged cell extracts were precipitated with anti-HA agorose (US28 or BILF1), precipitated proteins and whole cell lysates were analyzed by immunoblot with indicated antibodies. (C) 293T cells were transfected with the NFAT luciferase reporter cocktail and increasing amount of a plasmid containing wildtype kGPCR, EBV BILF1 or the BILF1 chimera (BILF1c) in which the carboxyl terminal tail and cytoplasmic loops of BILF1 were replaced with counterparts of kGPCR. NFAT activation was determined by luciferase assay at 30 hours post-transfection. Whole cell lysates were analyzed by immunoblotting for the expression of viral GPCRs (A and C).
(TIF)

S5 Fig The BILF1 chimera (BILF1c) interacts with SERCA2 and activates NFAT. (A) 293T cells were transfected with plasmids containing SERCA2 with BILF1 or BILF1c. Co-immunoprecipitation was performed with anti-HA-conjugated agarose (BILF1 or BILF1c). Precipitated proteins and whole cell lysates (WCL) were analyzed by immunoblotting. (B and C) HEK293T cells were transfected with the NFAT reporter cocktail and a plasmid containing BILF1c. At 24 h post-transfection cells were treated with EGTA (1–5 mM) or BAPTA-AM (10, 20 and 50 μM) for 5 h and NFAT activation was determined by luciferase reporter assay. (D) 293T cells were transfected with the NFAT reporter cocktail and a plasmid containing BILF1c. At 6 h post transfection cells were treated with the indicated compounds and NFAT activation was determined by luciferase reporter assay. Whole cell lysates were analyzed by immunoblotting for the expression of BILF1c (B-D).
(TIF)

S6 Fig Generation of KSHV BAC16 with kGPCR or kGPCRc revertant. (A-B) Gel electrophoresis of *Kpn*I- (A) or *Sbf*I-digested (B) BAC16 constructs (WT, ΔvGPCR, kGPCR revertant clone 1–5 and kGPCRc revertant clone 1–5). (C) PCR products were amplified from the kGPCR locus of KSHV and were analyzed by agarose gel electrophoresis. (D) iSLK cells carrying wild-type and kGPCR-deficient BAC16, revertant BAC16 with wild-type kGPCR or signaling defective kGPCRc were transfected with a plasmid containing EGFP-NFAT1 (1–460). At 12 hours post-transfection, cells were induced with sodium butyrate (1 mM) and doxycycline (0.5 μg/ml) for 48 hours. Whole cell lysates were analyzed by immunoblotting with indicated antibodies.
(TIF)

S7 Fig kGPCR activates NFAT in a paracrine mechanism. (A-C) Human Kaposi’s sarcoma tumors were analyzed by immunohistochemistry staining with antibodies against LANA (A), COX-2 (B) and RCAN1 (C). Images of low magnification were collected. Scale bars denote 50 μm. (D) HUVEC cells were incubated with conditioned medium from HUVEC/Vec (control) or HUVEC/kGPCR cells, with a control (CTL) or anti-IL-8 antibody (0.5 μg), for indicated time. Total RNA was extracted and analyzed by qRT-PCR with primers for COX-2 and RCAN1.
(TIF)
