# Supplementary figures and images for: Herpesviral G Protein-Coupled Receptors Activate NFAT to Induce Tumor Formation via Inhibiting the SERCA Calcium ATPase
Source: PLoS Pathog. 2015 Mar 26;11(3):e1004768. doi: 10.1371/journal.ppat.1004768 (PMC4374719; doi:10.1371/journal.ppat.1004768)

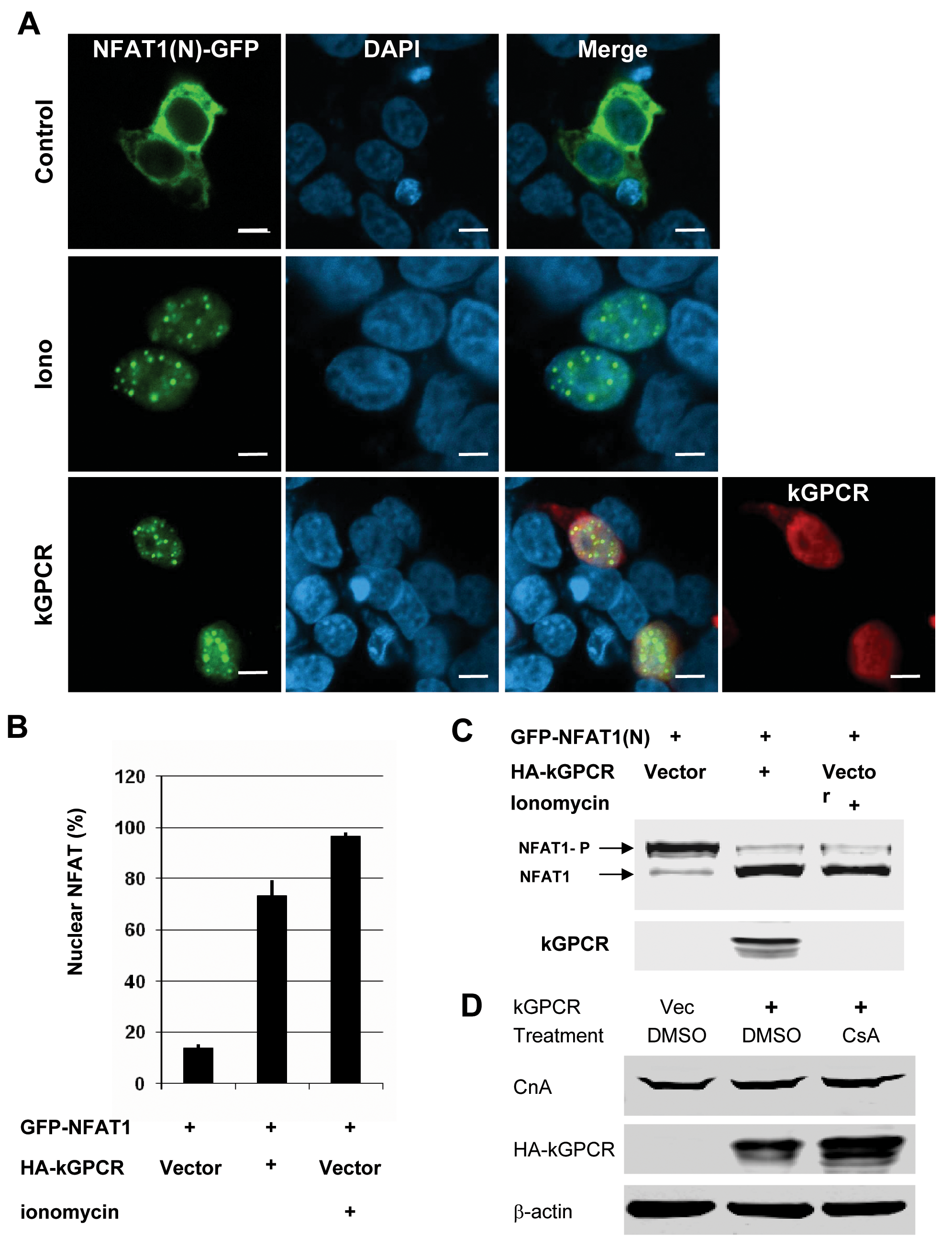

Supplement: S1 Fig — (A) HEK 293T cells were transfected with plasmids containing EGFP-NFAT1(N) and kGPCR. At 24 h post-transfection, cells were treated with ionomycin (Iono) (1 μM) and then fixed, stained and analyzed by immunofluorescence microscopy. Representative images were shown. Scale bar, 20 μm. (B) 293T cells were transfected with a plasmid containing EGFP-N1(N) without or with a plasmid containing kGPCR. For 293T cells transfected with the plasmid containing EGFP-NFAT1(N), cells were treated with vehicle (DMSO) or ionomycin (1 μM) for 6 hours. Nuclear NFAT1 was counted with fluorescence microscope. (C and D) Transfection and NFAT activation by ionomycin (C) or cyclosporine A (CsA, D) were carried out in (B). Whole cell lysates were analyzed by immunoblotting with indicated antibodies. (TIF) [file ppat.1004768.s001.tif]

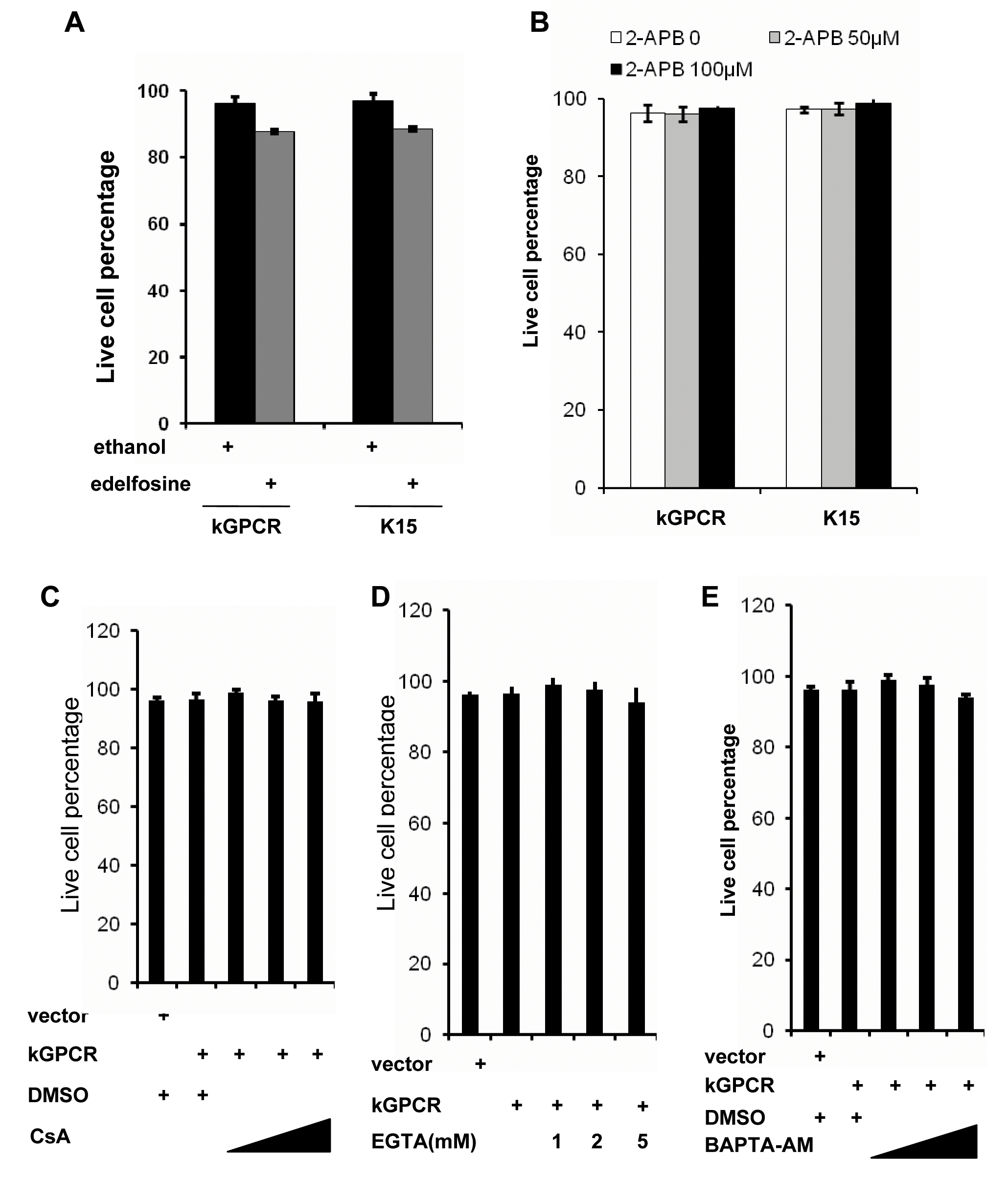

Supplement: S2 Fig — 293T cells were transfected with plasmids of NFAT reporter and that containing kGPCR. At 6 h post-transfection cells were treated with edelfosine (10 μM) (A), 2-APB (50 and 100 μM) (B) or cyclosporine A (CsA, 2, 20 and 200 nM) (C). At 24 hours, cells were treated with EGTA (1–5 mM) (D) or BAPTA-AM (10, 20 and 50 μM) (E) for 5 hours. Cell viability was determined by trypan blue staining. (TIF) [file ppat.1004768.s002.tif]

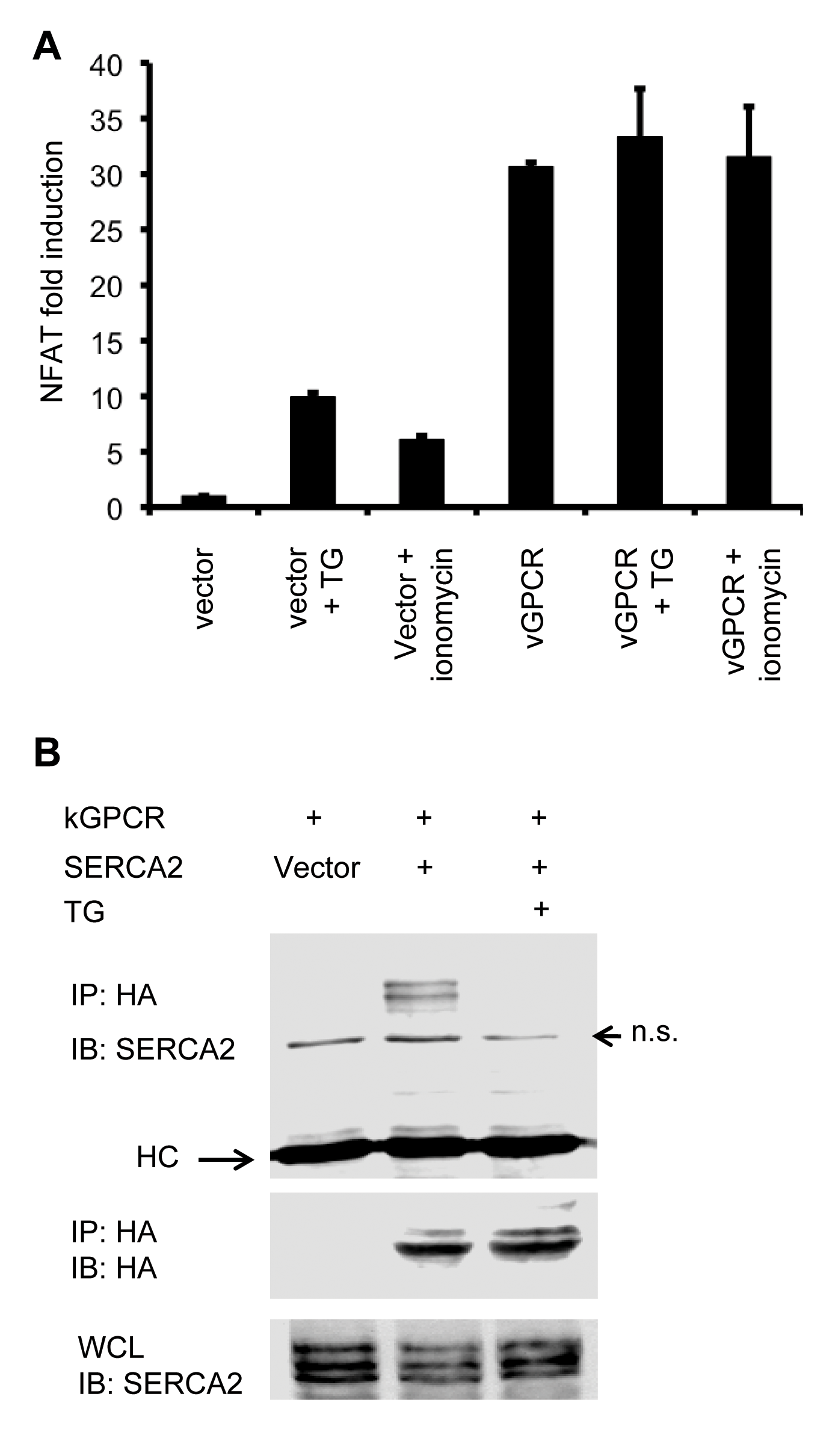

Supplement: S3 Fig — (A) HEK293T cells were transfected with the NFAT reporter cocktail and a plasmid containing KSHV GPCR. At 20 h post-transfection cells were treated with 1μM of TG or ionomycin for 6 h and NFAT activation was determined by luciferase reporter assay. (B) 293T cells were transfected with plasmids containing indicated genes and, 24 h post-transfection, cells were treated with vehicle (DMSO) or TG (1 μM) for 6 h. Co-immunoprecipitation and immunoblot were carried out as in (B). HC, IgG heavy chain. (TIF) [file ppat.1004768.s003.tif]

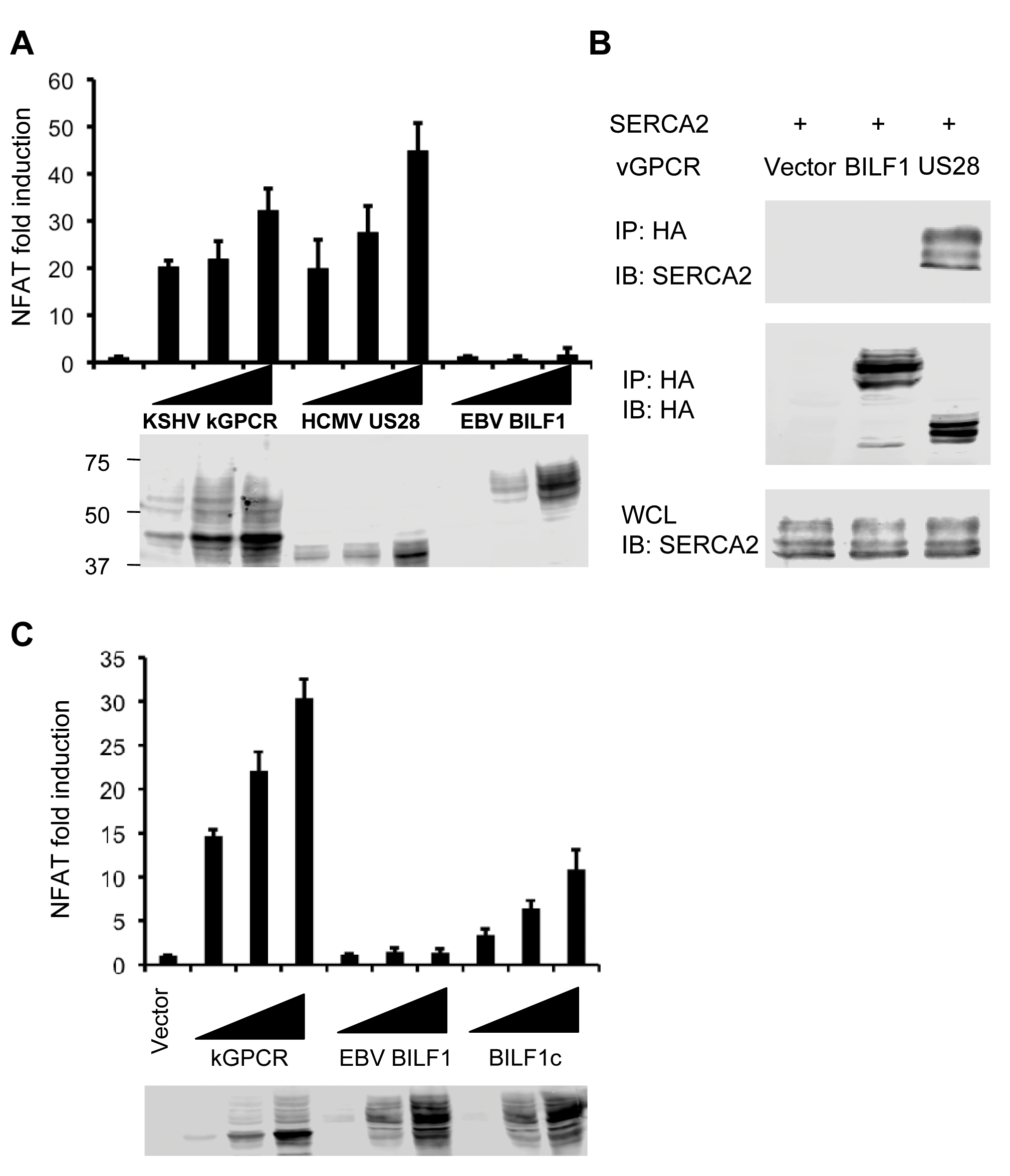

Supplement: S4 Fig — (A) 293T cells were transfected with the NFAT reporter cocktail and increasing amount of plasmids containing KSHV kGPCR, HCMV US28 and EBV BILF1. NFAT activation was determined by luciferase reporter assays. (B) 293T cells were transfected with plasmids containing SERCA2 and US28 or BILF1. Centrifuged cell extracts were precipitated with anti-HA agorose (US28 or BILF1), precipitated proteins and whole cell lysates were analyzed by immunoblot with indicated antibodies. (C) 293T cells were transfected with the NFAT luciferase reporter cocktail and increasing amount of a plasmid containing wildtype kGPCR, EBV BILF1 or the BILF1 chimera (BILF1c) in which the carboxyl terminal tail and cytoplasmic loops of BILF1 were replaced with counterparts of kGPCR. NFAT activation was determined by luciferase assay at 30 hours post-transfection. Whole cell lysates were analyzed by immunoblotting for the expression of viral GPCRs (A and C). (TIF) [file ppat.1004768.s004.tif]

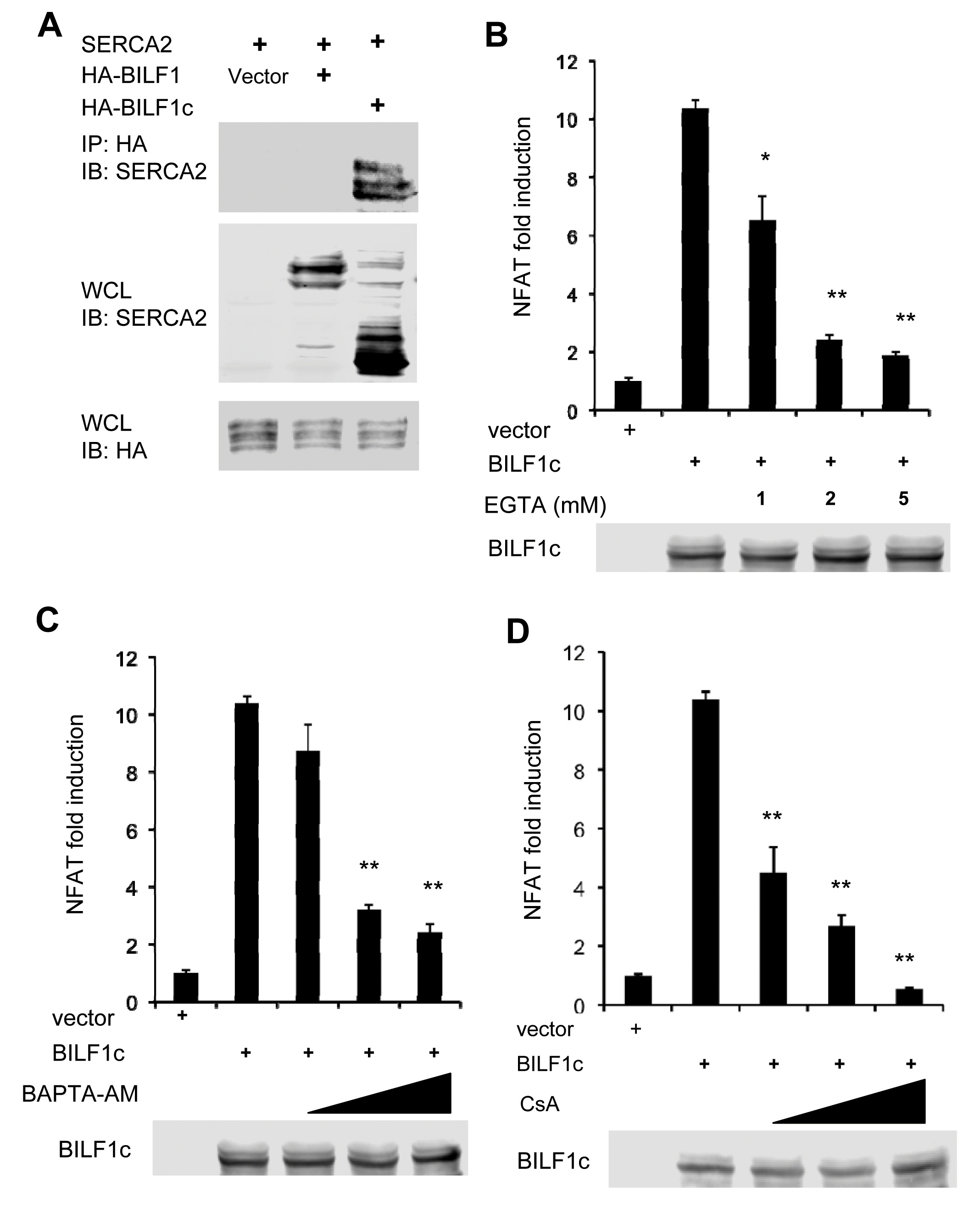

Supplement: S5 Fig — (A) 293T cells were transfected with plasmids containing SERCA2 with BILF1 or BILF1c. Co-immunoprecipitation was performed with anti-HA-conjugated agarose (BILF1 or BILF1c). Precipitated proteins and whole cell lysates (WCL) were analyzed by immunoblotting. (B and C) HEK293T cells were transfected with the NFAT reporter cocktail and a plasmid containing BILF1c. At 24 h post-transfection cells were treated with EGTA (1–5 mM) or BAPTA-AM (10, 20 and 50 μM) for 5 h and NFAT activation was determined by luciferase reporter assay. (D) 293T cells were transfected with the NFAT reporter cocktail and a plasmid containing BILF1c. At 6 h post transfection cells were treated with the indicated compounds and NFAT activation was determined by luciferase reporter assay. Whole cell lysates were analyzed by immunoblotting for the expression of BILF1c (B-D). (TIF) [file ppat.1004768.s005.tif]

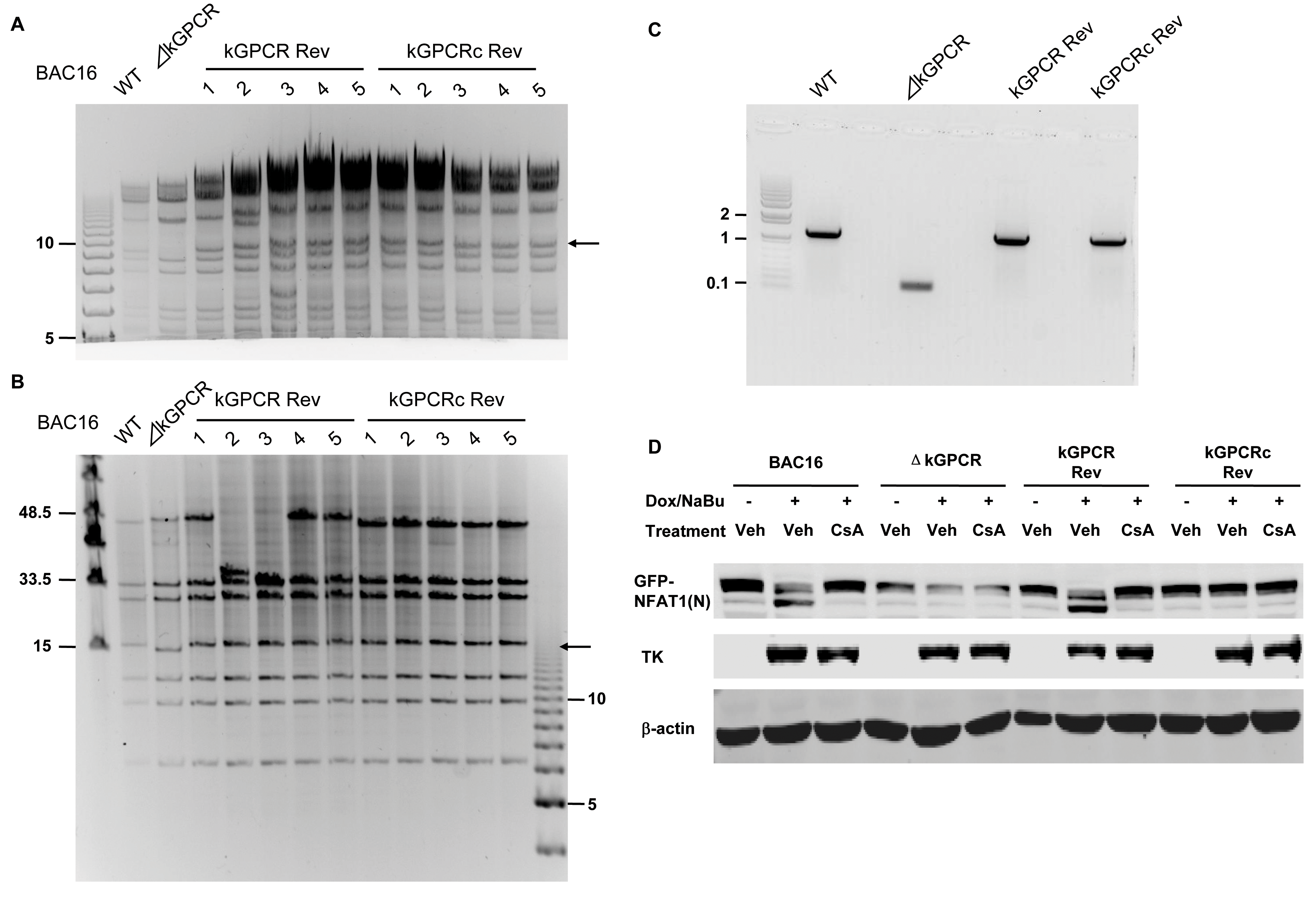

Supplement: S6 Fig — (A-B) Gel electrophoresis of KpnI- (A) or SbfI-digested (B) BAC16 constructs (WT, ΔvGPCR, kGPCR revertant clone 1–5 and kGPCRc revertant clone 1–5). (C) PCR products were amplified from the kGPCR locus of KSHV and were analyzed by agarose gel electrophoresis. (D) iSLK cells carrying wild-type and kGPCR-deficient BAC16, revertant BAC16 with wild-type kGPCR or signaling defective kGPCRc were transfected with a plasmid containing EGFP-NFAT1 (1–460). At 12 hours post-transfection, cells were induced with sodium butyrate (1 mM) and doxycycline (0.5 μg/ml) for 48 hours. Whole cell lysates were analyzed by immunoblotting with indicated antibodies. (TIF) [file ppat.1004768.s006.tif]

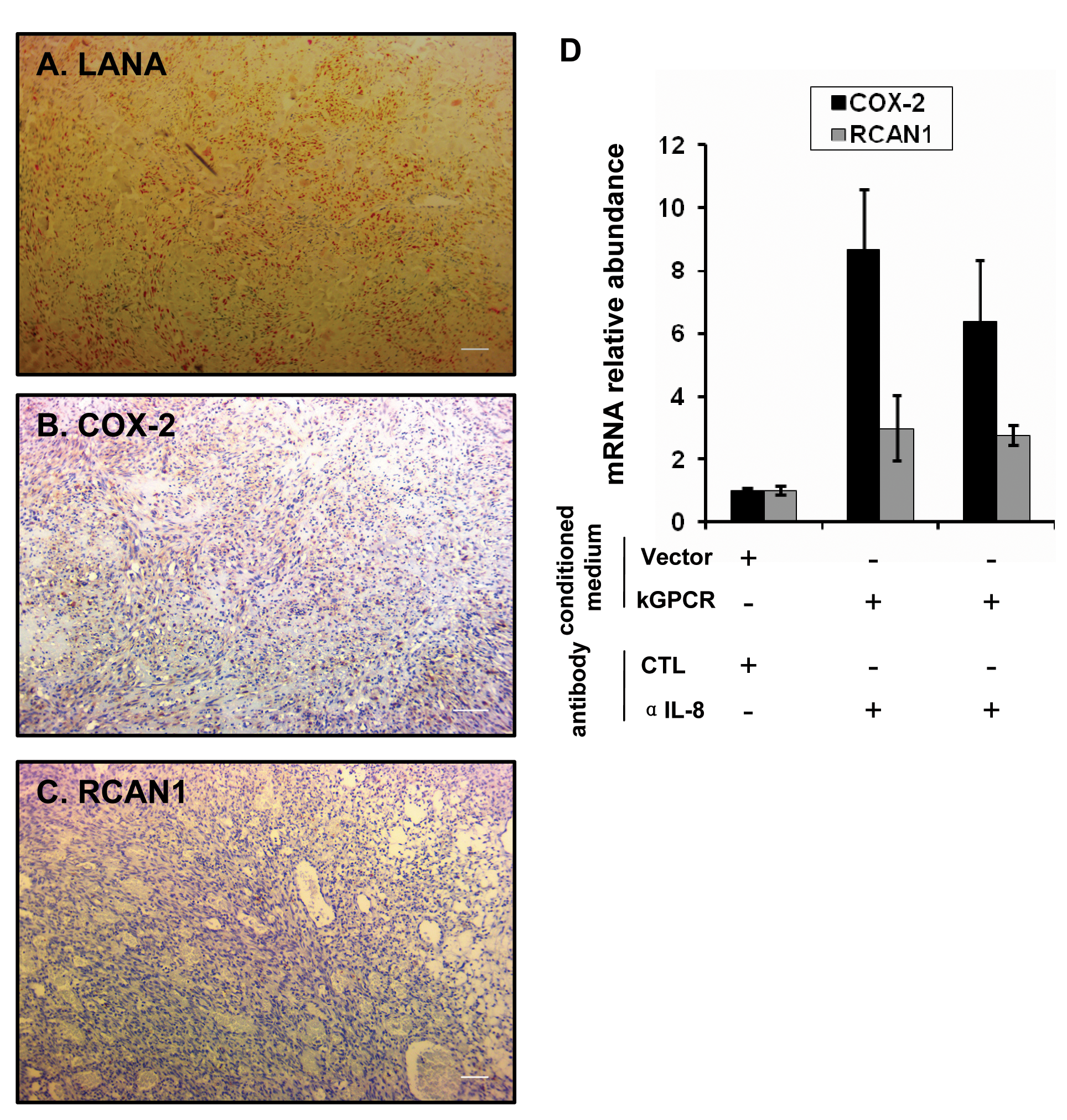

Supplement: S7 Fig — (A-C) Human Kaposi’s sarcoma tumors were analyzed by immunohistochemistry staining with antibodies against LANA (A), COX-2 (B) and RCAN1 (C). Images of low magnification were collected. Scale bars denote 50 μm. (D) HUVEC cells were incubated with conditioned medium from HUVEC/Vec (control) or HUVEC/kGPCR cells, with a control (CTL) or anti-IL-8 antibody (0.5 μg), for indicated time. Total RNA was extracted and analyzed by qRT-PCR with primers for COX-2 and RCAN1. (TIF) [file ppat.1004768.s007.tif]
